# Fucoidan and alginate from brown seaweeds: extraction, structural diversity, biocompatibility, biodegradability, and biomedical applications

**DOI:** 10.3389/fpls.2026.1845395

**Published:** 2026-06-10

**Authors:** Mostafa M. El-Sheekh, Samar S. Alkafaas, Eman Bases, Feifei Zhu, Xu Yan, Wesam E. Yousuf, Shimaa M. El Shafay, Rania A. El-shenody, Amira M. Abu-Resha, Alaa E. Etman, Dorya E. Essa, Abdullah A. Saber, Shuhao Huo

**Affiliations:** 1 Botany and Microbiology Department, Faculty of Science, Tanta University, Tanta, Egypt; 2 Molecular Cell Biology Unit, Division of Biochemistry, Department of Chemistry, Faculty of Science, Tanta University, Tanta, Egypt; 3 School of Food and Biological Engineering, Jiangsu University, Zhenjiang, China; 4 Biochemistry Division, Chemistry Department, Faculty of Science, Tanta University, Tanta, Egypt; 5 Botany and Microbiology Department, Faculty of Science, Menoufia University, Shibin Elkoum, Egypt; 6 Biology Department, College of Science, Imam Mohammad Ibn Saud Islamic University (IMSIU), Riyadh, Saudi Arabia

**Keywords:** alginate, biocompatibility, biodegradability, brown seaweeds, drug delivery systems, extraction techniques, fucoidan, marine polysaccharides

## Abstract

Brown seaweeds are among the richest marine sources of structurally diverse polysaccharides, especially fucoidan and alginate, which have attracted significant attention as multifunctional biomaterials for pharmaceutical, biomedical, and regenerative medicine applications. This review provides a comprehensive overview of extraction and purification methods, including conventional chemical, enzymatic, microwave-assisted, and ultrasound-assisted techniques, discussing how these methods affect yield, purity, molecular weight distribution, sulfation patterns, and structural integrity. An in-depth examination of the chemical structure of fucoidan and alginate highlights the importance of monosaccharide composition, sulfation degree, M/G ratio, molecular weight, and block arrangement in influencing bioactivity, gelation, biodegradability, and interactions with biological systems. The review thoroughly evaluates biocompatibility and biodegradation mechanisms, focusing on the roles of impurities, crosslinking density, oxidation levels, enzymatic degradation pathways, and environmental factors. It also summarizes recent advances in fucoidan- and alginate-based formulations, including hydrogels, nanoparticles, films, nanofibers, microspheres, sponges, injectable gels, and composite scaffolds, as well as key drug delivery mechanisms like ionotropic gelation, diffusion-controlled release, pH-responsiveness, mucoadhesion, and stimuli-responsive behaviors. By combining structural insights with emerging biomedical applications, this review highlights the remarkable versatility of fucoidan and alginate as next-generation marine biomaterials and explores their expanding potential in drug delivery, tissue engineering, wound healing, and multifunctional therapeutic systems.

## 1 Introduction

Marine macroalgae have long been considered a productive source of structurally diverse polysaccharides with significant biological and technological value (Biris-Dorhoi et al., 2020; Lesco et al., 2025). Among these biopolymers, fucoidan and alginate, which are predominantly obtained from brown seaweeds (Phaeophyceae), represent two of the most extensively studied marine-derived materials due to their abundance, biocompatibility, and broad applicability in biomedical and pharmaceutical fields (Bilal and Iqbal, 2019; Ghalsasi et al., 2025). Their chemical architecture, ease of modification, and tunable physicochemical characteristics have positioned them as essential candidates for next-generation biomaterials and bioactive formulations (Chakrapani et al., 2025). Seaweeds are rich in a wide range of bioactive metabolites, including polysaccharides, polyphenols, flavonoids, pigments, proteins, and other phytochemicals. These phytochemicals contribute significantly to their diverse biological activities, such as antioxidant, antimicrobial, and anti-inflammatory effects (Mandal et al., 2025). Phenolic compounds, ranging from simple acids to complex flavonoids, play a key role in antioxidant activity by chelating metal ions and reducing free radical formation. In addition, seaweeds contain various bioactive substances such as terpenoids, phlorotannins, fatty acids, and amino acids, which exhibit antimicrobial effects. They are also a valuable source of pigments like fucoxanthin and β-carotene, known for their ability to quench reactive oxygen species. These compounds can be efficiently extracted using different organic and inorganic solvents (Subbiah et al., 2023; Afrin F. et al., 2025). Polysaccharides are long-chain polymeric carbohydrates comprised of monosaccharide units that are connected by glycosidic linkages. Seaweed-derived polysaccharides possess complex chemical structures and diverse functional groups, which give them unique physicochemical properties and a wide range of biological activities. These compounds can interact with various molecules, including lipids, proteins, and microbiota, making them promising biopolymers for multiple applications (Saravana et al., 2018; Liu Z. et al., 2022). In recent decades, research has increasingly focused on brown algae due to their rich content of bioactive compounds exhibiting antimicrobial, anticancer, antioxidant, anti-inflammatory, antidiabetic, and antiparasitic properties. Key constituents, including phlorotannins, fucoxanthin, alginic acid, fucoidan, and laminarin, have been widely studied for their chemical composition and functional bioactivities (Remya et al., 2022a; Xie et al., 2024).

Fucoidan is a sulfated polysaccharide that is mainly composed of L-fucose and sulfate esters, often accompanied by varying amounts of galactose, mannose, xylose, and uronic acids (Alfinaikh et al., 2025; Ko et al., 2025). The structural heterogeneity of fucoidan, shaped by species differences, geographic factors, seasonal variations, and extraction conditions, has been directly linked to its diverse biological activities (Pereira and Valado, 2025; Zhang et al., 2025). Numerous studies demonstrate that fucoidan has anticoagulant, anti-inflammatory, antiviral, antioxidant, antitumor, and immunomodulatory effects, with its molecular weight and degree and pattern of sulfation serving as key determinants of bioactivity and cellular interactions (Alfinaikh et al., 2025; Farrag, 2025; Prabhu et al., 2025). This strong structure–function relationship continues to drive interest in optimizing fucoidan for advanced therapeutic applications (Jayawardena et al., 2022). The seaweed farming industry has experienced significant global growth, with a valuation of approximately USD 5.9 billion in 2019 and a projected compound annual growth rate (CAGR) of 9.1% through 2027. Emerging markets are expected to reach nearly USD 11.8 billion by 2030 (Das et al., 2025), reflecting increasing interest in marine resources. Within this context, fucoidan has gained considerable scientific and industrial attention as a sulfated polysaccharide derived mainly from brown seaweeds (Iqbal et al., 2024). The increasing adoption of commercial seaweed as functional foods, cosmeceuticals, nutraceuticals, and pharmaceuticals is expected to propel the seaweed market growth. The European Union has also approved fucoidan extracts from *F. vesiculosus* and *U. pinnatifida* as novel foods for use in foods and food supplements. Several commercial fucoidans from *F. vesiculosus* and other brown algae species are marketed by well-known companies, including Sigma-Aldrich^®^, Algues, Mer and Marinova^®^ (Iqbal et al., 2024; Das et al., 2025).

Alginate, another significant polysaccharide from brown seaweeds, is made up of linear chains of β-D-mannuronic acid (M) and α-L-guluronic acid (G) (Rashedy et al., 2021; Alrata et al., 2025; El-Sheekh et al., 2025a). Its physicochemical properties, including viscosity, gel strength, porosity, and degradation behavior, are primarily dictated by the M/G ratio and the arrangement of M- and G-blocks, which in turn affect its interactions with divalent cations and its suitability for hydrogel formation. Due to its excellent biocompatibility, mild gelation mechanism, and chemical versatility, alginate has become widely used in drug delivery, wound healing, cell encapsulation, tissue engineering, and 3D bioprinting (Liu et al., 2025; Yadav et al., 2025).

Recent technological advancements have markedly improved the extraction, purification, and modification of fucoidan and alginate (Chadwick et al., 2025; Srivastava et al., 2025). Enzyme-assisted, ultrasound-assisted, microwave-assisted, and hybrid extraction techniques have enabled higher yields, greater structural preservation, and reduced processing time while minimizing chemical degradation (Mgoma et al., 2025; Stanek-Wandzel et al., 2025). Furthermore, chemical modifications, such as sulfation, phosphorylation, oxidation, and conjugation with bioactive molecules, have expanded their functional versatility, enhancing properties such as biodegradability, mucoadhesion, mechanical strength, and controlled release capabilities (Surendranath et al., 2022; Alfinaikh et al., 2025).

Despite their promise, challenges remain, including batch variability, incomplete structural characterization, the scalability of extraction techniques, and limited clinical translation. A major challenge to standardization and regulatory acceptance is the batch-to-batch diversity that results from variations in seaweed sources, natural factors and extraction procedures. This variability causes inconsistencies in molecular composition and biological activity. Addressing these gaps is essential for standardizing fucoidan- and alginate-based biomaterials and improving their reproducibility for pharmaceutical and biomedical applications. Therefore, this review provides an in-depth and updated overview of extraction strategies, structural features, biocompatibility, biodegradability, and advanced biomedical applications of fucoidan and alginate derived from brown seaweeds. By correlating structural attributes with functional performance, the review aims to support future development of marine polysaccharide–based biomaterials for drug delivery, regenerative medicine, and tissue engineering.

## 2 Marine-derived polysaccharides

The oceans contain a vast proportion of the planet’s natural resources, positioning marine-derived biomaterials as a sustainable and long-term source for diverse biotechnological and biomedical applications. These biomaterials are produced by a broad range of marine organisms, including algae, invertebrates, vertebrates, and microorganisms, and exhibit remarkable biochemical diversity. Among them, marine-derived polysaccharides have received significant scientific attention due to their wide spectrum of biological activities (Carrasqueira et al., 2025). These activities include antioxidant, anti-inflammatory, anticancer, antidiabetic, anti-obesity, and gut microbiota–modulating effects (de Jesus Raposo et al., 2015).

Marine biomaterials include a variety of natural polymeric substances, such as alginate, carrageenan, sulfated polysaccharides, chitosan, agar, fucoidan, and collagen, as well as bioactive compounds including fatty acids, carotenoids, sterols, phlorotannins, squalene, mycosporine-like amino acids (MAAs), minerals, and vitamins (Krishnamurthi et al., 2024; Kumar et al., 2023; Zhu et al., 2024; Carrasqueira et al., 2025; Chellapandian et al., 2025). Algal biomass is one of the most abundant marine resources, providing a diverse range of compounds with promising applications in regenerative medicine, drug delivery, and disease management. Numerous natural biopolymers with intrinsic antibacterial and anti-inflammatory activities have been isolated from marine organisms (Saudagar et al., 2019). Notably, polysaccharides like carrageenan, fucoidan, alginate, and agar have gained increasing importance in medical and pharmaceutical research. Their physicochemical properties can be adjusted through biochemical modification, enabling enhanced drug affinity, loading efficiency, and controlled release. Additionally, chemical derivatization or blending with other natural polymers can further improve their therapeutic performance (Carrasqueira et al., 2025).

Marine-derived polysaccharides (MDP) are highly valued for their versatility, biodegradability, and biocompatibility, making them useful in wound dressings, tissue engineering scaffolds, and advanced drug delivery systems (Nayak et al., 2022). Their broad availability, biological importance in marine organisms, and cost-effective extraction methods further enhance their growing relevance (Jim et al., 2025). The pharmacological activities of MDP are largely determined by structural features such as monosaccharide composition, glycosidic linkages, and the degree of sulfation or acetylation. For example, the high sulfation level of fucoidan explains its strong immunomodulatory and anticoagulant properties. Conversely, the acetylated amino groups in chitosan are crucial for lipid-lowering effects and antimicrobial activity (Ghosh et al., 2021). Structural parameters, including sulfation patterns, degree of acetylation, and backbone configuration, significantly influence the bioactivities of major marine polysaccharides such as fucoidan (highly sulfated fucan), alginate (rich in mannuronic and guluronic acids), laminarin (β-glucan), carrageenan (sulfated galactan), and chitosan (deacetylated chitin) (Dongre, 2022; Jim et al., 2025).

## 3 Overview of brown seaweeds

Brown seaweeds (Phaeophyceae) are a diverse group of marine macroalgae characterized by their distinctive coloration, ranging from olive green to golden brown (Nguyen and Tran, 2024). This pigmentation is primarily attributed to the xanthophyll fucoxanthin, a dominant accessory pigment that masks other photosynthetic pigments such as chlorophylls *a* and *c*. Members of this group display extensive morphological diversity, spanning from small filamentous species to giant kelps that can exceed 50 meters in length, with the majority thriving in intertidal and subtidal habitats (Remya et al., 2022a; Remya et al., 2022b). Nearly all brown algae are exclusively marine and play essential ecological and economic roles. They are widely utilized as sources of food, fertilizers, and a broad range of bioactive compounds (El-Manaway and Rashedy, 2022).

**Figure 1:**
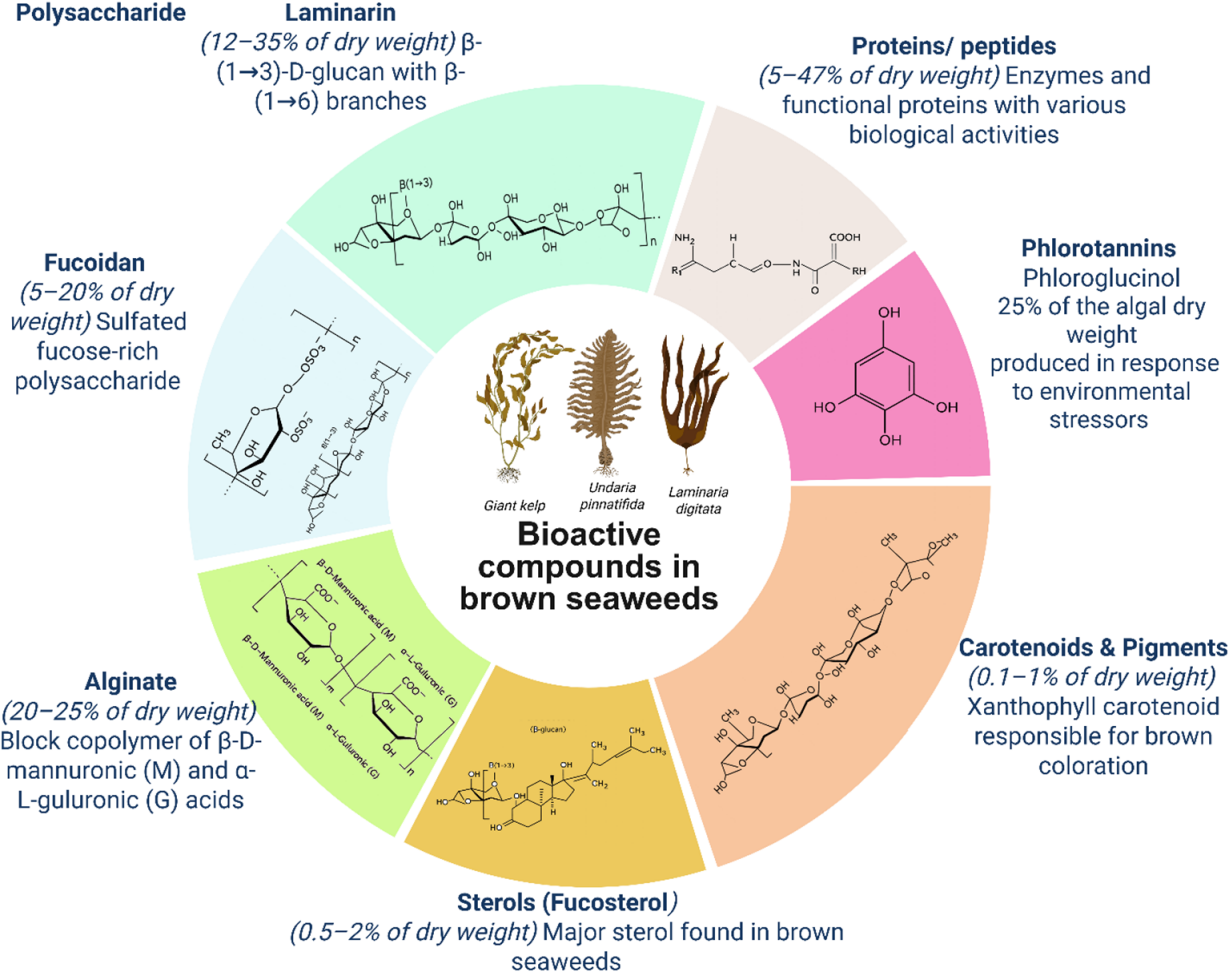
Key bioactive components and biochemical composition of brown seaweeds.

Brown seaweeds are particularly rich in biologically active components, including polysaccharides (like alginate, fucoidan), proteins (such as proteins and bioactive peptides), polyphenols (notably phlorotannins), carotenoids (including fucoxanthin), phytosterols (such as fucosterol), and n-3 long-chain polyunsaturated fatty acids (like eicosapentaenoic acid), as shown in **Figure 1** (Olsthoorn et al., 2021). These bioactive molecules underpin their increasing relevance in pharmaceutical, nutraceutical, and cosmeceutical applications (Singh and Negi, 2025).

In addition, brown seaweeds contain substantial levels of polyphenols, peptides, carotenoids, and structurally diverse polysaccharides (Ismail et al., 2023). Crude polysaccharide extracts from brown algae have been reported to exhibit stronger antioxidant activity and higher sulfate and polyphenol content than those from green or red seaweeds. Furthermore, polysaccharides isolated from brown algae show notable anticoagulant and antithrombotic properties, suggesting their potential use as functional ingredients in medicinal formulations (Fauziee et al., 2021).

### 3.1 Bioactive compounds in brown seaweeds and their potential preventive and therapeutic effects

#### 3.1.1 Proteins/peptides

Brown seaweeds contain approximately 5 to 47% protein (dry weight), and the peptides derived from these proteins exhibit a variety of bioactive properties (Echave et al., 2022). Peptides are typically defined as protein fragments composed of 3 to 40 amino acids. Enzymatic hydrolysis of seaweed proteins can produce peptide-enriched extracts, and interestingly, the amino acid sequences within intact proteins are not always biologically active until they are released in peptide form (Pliego-Cortés et al., 2020; Jang et al., 2024). Several of these peptides function as natural antihypertensive agents by inhibiting angiotensin-I converting enzyme (ACE I), an enzyme involved in the production of the potent vasoconstrictor angiotensin II and the stimulation of aldosterone secretion. Inhibition of ACE I lowers angiotensin II formation, which reduces blood pressure through decreased vasoconstriction and reduced sodium and water retention. Examples of such activity include peptides isolated from *Undaria pinnatifida*, which have demonstrated ACE-inhibitory effects, and protein hydrolysates obtained from *Sargassum ilicifolium, which* exhibit notable antioxidant activity (Echave et al., 2021; Jang et al., 2024).

#### 3.1.2 Polyphenols

Polyphenols are important bioactive metabolites produced by brown seaweeds in response to environmental stressors such as ultraviolet (UV) radiation, physical injury, and climate fluctuations (Fernando et al., 2022). These compounds exhibit a broad spectrum of biological activities, including antioxidant, antiviral, anticancer, antibacterial, antidiabetic, neuroprotective, and antiallergic effects (Chojnacka et al., 2021; Davidova et al., 2024). Among the various polyphenolic constituents, phlorotannins are the most common class in Phaeophyta (brown algae) and are largely responsible for many of these bioactivities (Murray et al., 2018; Wekre et al., 2019).

#### 3.1.3 Phlorotannins

Brown seaweeds (Phaeophyta) contain a distinctive class of polyphenolic compounds known as phlorotannins, which may represent up to 25% of the algal dry weight. Phlorotannins are oligomers or polymers of phloroglucinol (1,3,5-trihydroxybenzene), formed through phenyl (C–C) bonds, ether (C–O–C) linkages, or combinations of both (Ford et al., 2020; Wekre et al., 2022). These compounds are highly hydrophilic and have a wide molecular weight range, from approximately 126 Da to 650 kDa. Within seaweeds, phlorotannins play key physiological roles, contributing to cell wall structure, providing protection against ultraviolet (UV) radiation, and functioning as chemical defenses against marine herbivores (Cotas et al., 2020; Shrestha et al., 2021).

Based on the types of linkages between phloroglucinol units, phlorotannins are categorized into six major groups: fuhalols, phlorethols, fucols, fucophlorethols, eckols, and carmalols. Among these, dieckol, a prominent phlorotannin isolated from the brown alga *Ecklonia cava* (paddle weed, Kajime), has been shown to protect against high-glucose–induced oxidative stress in human umbilical vein endothelial cells (Lopes et al., 2016).

#### 3.1.4 Polysaccharide

Brown seaweed polysaccharides (BSP) constitute approximately (12% to 56%) of the dry weight and have received considerable attention due to their wide-ranging biological and pharmacological roles, including prebiotic, antioxidant, anticancer, anti-inflammatory, antiphotoaging, and immunomodulatory effects (Shannon and Abu-Ghannam, 2019; Yao et al., 2022; Yao et al., 2023). Alginic acid, sulfated fucans, laminarin, cellulose, mannitol, and various β-glucans are the main carbohydrate components of brown seaweeds (Koh et al., 2019).

Alginic acid is a linear anionic polysaccharide composed of repeating units of β-D-mannuronic acid (ManA) and α-L-guluronic acid (GulA) connected by 1,4-glycosidic linkages (Ammar et al., 2018). In nature, alginic acid typically occurs as its mineral salts, collectively referred to as alginates (e.g., sodium alginate). Due to their biocompatibility, gel-forming capability, and multifunctional physicochemical properties, alginates are widely used in the food industry, pharmaceutical formulations, wound dressings, drug delivery systems, and cosmetics (Guo et al., 2020).

A second major group of BSP is the fucose-containing sulfated polysaccharides (FCSPs), which encompass sulfated fucans and fucoidans (Ale et al., 2011b). The backbone of sulfated fucans is typically made up mostly of fucose residues, which can be altered by the addition of additional fucose units, galactose, xylose, sulfate, or acetyl groups (Alfinaikh et al., 2025). Fucoidans, in contrast, represent a broader and more structurally diverse class of fucose-rich polysaccharides. Their backbones often include uronic acids, galactose, mannose, and other sugars, contributing to their complex architectures and diverse bioactivities, such as anticoagulant, antiviral, antioxidant, and anticancer functions (Benslima et al., 2021; Sanniyasi et al., 2023).

Laminarins, the primary storage polysaccharides of brown seaweeds, are low molecular weight β-D-glucans made up mostly of β-(1→3)-linked glucose units branched at the O-6 position (Graiff et al., 2016). In some cases, residues of 6-linked glucose can connect chains of β-(1→3)-glucans. There are two main structural forms of laminarin: the M-chain, which has a mannitol group at the reducing end, and the G-chain, which ends with a free glucose residue. Laminarin’s diverse biological effects, including immunomodulatory, prebiotic, and antioxidant properties, are facilitated by its structural heterogeneity (Kadam et al., 2015; Karuppan Perumal et al., 2023; Sanniyasi et al., 2023; Jang et al., 2024).

#### 3.1.5 Carotenoids and pigments

Brown seaweeds have four major pigments [chlorophylls (a and c), β-carotene, fucoxanthin, and lycopene] with their levels influenced by species, season, depth, and extraction methods. Chlorophylls, especially chlorophyll c, play vital roles in photosynthesis and light harvesting. β-Carotene functions as a provitamin A compound important for vision and tissue health, while fucoxanthin, the predominant carotenoid in brown algae, exhibits powerful antioxidant, anticancer, antidiabetic, and anti-inflammatory properties. Lycopene, although lacking provitamin A activity, is a potent antioxidant with greater singlet oxygen quenching ability than β-carotene and α-tocopherol, highlighting the diverse bioactive potential of brown seaweed pigments (Mtaki et al., 2020; Ismail et al., 2023).

Carotenoids are pigments found in brown seaweeds that have important biological roles, especially as antioxidants. They serve as scavengers of reactive oxygen species (ROS), thereby lowering oxidative stress within cells. Structurally, carotenoids are divided into two main groups: carotenes (hydrocarbon carotenoids) and xanthophylls (oxygenated derivatives) (Savira et al., 2021). Among these, fucoxanthin is the most common carotenoid in brown seaweeds and is part of the xanthophyll group. Its antioxidant activity is due to its ability to donate electrons and neutralize free radicals, preventing oxidative chain reactions. Fucoxanthin extracted from *Padina australis* and other brown seaweeds has been shown to have strong antioxidant properties. Because of its natural origin and bioactivity, fucoxanthin is a promising candidate to replace or supplement synthetic antioxidants in the pharmaceutical, nutraceutical, and food industries (Osório et al., 2020; Lourenço-Lopes et al., 2022).

#### 3.1.6 Sterols

Sterols represent a large class of lipid-derived compounds that function as essential components of cellular membranes and as precursors for hormone biosynthesis in both aquatic and terrestrial organisms (Hartmann, 2003). While red and green algae are the main sources of cholesterol and its derivatives, brown seaweeds are especially rich in fucosterol and related sterol alternatives. These marine sterols exhibit a wide range of biological actions, including antioxidant, anticancer, antimicrobial, and antiulcerative effects (Torres P. et al., 2019; Berneira et al., 2021).

## 4 Brown Seaweed Polysaccharides

### 4.1 Alginate

Alginate is a natural polysaccharide found in brown seaweeds primarily in the form of alginic acid. It is considered a major structural component of the cell walls in numerous species, including *Ascophyllum nodosum*, *Macrocystis pyrifera*, *Durvillaea antarctica*, *Sargassum turbinaroides*, *Lessonia nigrescens*, *Ecklonia maxima*, and several *Laminaria* species (King, 2022). In addition to brown algae, alginate can also be biosynthesized by certain bacteria, particularly Pseudomonas and Azotobacter. Microbial alginates exhibit distinct structural and physicochemical properties compared to algal alginates, mainly due to variations in monomer composition and arrangement (Hay et al., 2013). For instance, Pseudomonas aeruginosa produces alginate associated with biofilm formation, whereas Azotobacter vinelandii produces alginate with higher guluronic acid content, resulting in more rigid polymer structures (Bouzenad et al., 2025; Javeed et al., 2026). Chemically, alginate is a carbohydrate polymer composed of repeated units of monosaccharide. Within algal tissues, it occurs as water-insoluble salts of alginic acid, typically associated with magnesium, sodium, potassium, and calcium ions, which contribute to cell wall strength and rigidity. The polymer consists of linear, unbranched chains formed through 1,4-glycosidic linkages between β-D-mannuronic acid (M) and α-L-guluronic acid (G) residues (Lomartire and Gonçalves, 2022; Pai et al., 2023). The main structure of alginates is composed of two monomeric units: β-(1,4) linked d-mannuronic acid (ManAp or M) with 4C1 ring conformation and α-(1,4)-linked l-guluronic acid (GulAp or G) with 1C4 ring conformation (Ching et al., 2017). The physicochemical characteristics of alginate are affected by the ratio, sequence, and spatial arrangement of these monomers, as well as by its molecular weight and the type of counter-ions present (Mamede et al., 2023).

#### 4.1.1 Extraction methods of alginate polysaccharide

Alginates can be extracted from brown seaweeds using various techniques, with conventional chemical extraction being the most common, as shown in **Table 1**. In this process, fresh seaweed is washed, dried, and ground into a fine powder (Łabowska et al., 2019; Saji et al., 2022). The biomass is then soaked in an organic solvent, usually ethanol, to remove lipids, pigments, and polyphenols. After this purification step, an acid or alkaline solution is added to break down the cell wall. Next, sodium carbonate (Na_2_CO_3_) or sodium hydroxide (NaOH) is added to adjust the pH of the seaweed–water mixture to around 9-10, converting insoluble alginic acid into water-soluble alginate salts (Saji et al., 2022). Once dissolved, alginate can be precipitated using one of three established methods: the sodium alginate pathway, the calcium alginate pathway, or the alginic acid pathway, with the sodium alginate pathway being the most widely used.

**Table 1:** Extraction and purification of fucoidan and alginate polysaccharides from brown algae.

Algal name	Algal polysaccharide	Extraction method	Purification method	Reference
*Fucus vesiculosus*	Fucoidan	Microwave-assisted extraction (MAE) under optimized conditions	Ethanol fractionation, desalting	(Rodriguez-Jasso et al., 2011)
*Sargassum muticum*	Fucoidan	Ultrasound-Assisted Extraction (UAE), cavitation disrupts cell wall	Ethanol precipitation, dialysis	(Flórez-Fernández et al., 2017)
*Fucus evanescens*	Fucoidan	Ultrasound-Assisted Extraction (UAE)	Column chromatography, dialysis	(Hmelkov et al., 2018)
*Sargassum horneri*	Fucoidan	Enzyme-assisted extraction (AMG, Celluclast, Viscozyme, Alcalase)	Chromatography, desalting	(Sanjeewa et al., 2017)
*Saccharina latissima & Alaria esculenta*	Fucoidan	Chemical fucoidan extraction & Enzymatic fucoidan extraction	Alginate removal with 2% CaCl_2_ precipitation + centrifugation, Fucoidan recovery with ethanol (80%) precipitation.	(Rhein-Knudsen et al., 2023)
*Fucus vesiculosus* *; Ascophyllum nodosum*	Fucoidan	Dilute acid extraction under moderate heat	Membrane filtration + Anion-exchange chromatography	(Usov et al., 2022)
*Polycladia Myrica*	Fucoidan	McIlvaine’s buffer extraction	Alginate removal by CaCl_2_ precipitation and vacuum filtration Fucoidan purification by ethanol precipitation (2 volumes, overnight at 4 °C), centrifugation (1000 × g, 15 min), drying at 50 °C	(Abu-Resha et al., 2025)
*Polycladia Myrica*	Fucoidan	Ethanol defatting, acetone wash, hot water extraction at controlled pH and temperature	Fucoidan: ethanol precipitation (70%, overnight at 4 °C); Alginate: CaCl_2_ precipitation (1% at 4 °C), followed by centrifugation and drying	(El-Sheekh et al., 2025b)
*Sargassum ilicifolium*	Fucoidan	Solvent defatting → Acid extraction (HCl) → Ethanol precipitation	Ion-exchange chromatography (Q- Sepharose)	(Lakshmanan et al., 2022)
*Fucus vesiculosus*	Alginate	Ultrasound-Assisted Extraction (UAE) Conventional Extraction	Centrifugation → precipitation with NaCl and isopropanol → overnight cooling → filtration → drying at 35 °C.	(Ummat et al., 2024)
*Laminaria digitata*	Alginate	Sodium carbonate extraction (25–60 °C)	Filtration + Sodium alginate precipitation (ethanol)	(Fertah et al., 2017)
*Sargassum siliquosum*	Alginate	Acid pre-treatment → Alkaline extraction (Na_2_CO_3_)	Precipitation with ethanol / CaCl_2_ / HCl → drying, milling	(Chee et al., 2011)
*Durvillaea antarctica*	Alginate	Alkaline extraction	Ethanol precipitation	(Caballero et al., 2021)
*Sargassum cymosum*	Alginate	Alkaline extraction (Na_2_CO_3_, pH 10)	Ethanol precipitation	(Nogueira et al., 2022)
*Sargassum binderi, Turbinaria ornata*	Alginate	Ultrasound-Assisted Extraction (UAE)	Precipitation (ethanol / CaCl_2_)	(Youssouf et al., 2017)
*Sargassum muticum*	Alginate	Alkali-assisted extraction with ethanol precipitation	Ethanol precipitation	(Mazumder et al., 2016)
*Sargassum angustifolium*	Alginate	sequential acid-base extraction with ethanol precipitation,	dissolve in water → Re-precipitate with ethanol → Wash with acetone → Centrifuge → Dialyze → Lyophilize	(Behfar et al., 2022)
*Sargassum natans*	Alginate	Acid pre-treatment → Alkaline extraction → Calcium precipitation → Ethanol purification	Bleaching → Precipitation → Freeze-drying → Powdering	(Mohammed et al., 2020)
*Ecklonia maxima*	Alginate	Ultrasound-Assisted Extraction at pH 8–10, 50–60 °C, with or without alkaline cellulase (E:S 0–1%) → Extraction kinetics modeled	Not reported; alginate was collected as crude extract without further purification	(van Sittert et al., 2024)
*Dictyota mertensii*	Alginate	Extraction optimized by Response Surface Methodology (Na_2_CO_3_ treatment, statistical design and validation)	Alginate recovered after extraction	(de Oliveira Queiroz et al., 2024)
*Sargassum wightii*	Alginate	Drying → CaCl2 → Formaldehyde → HCl → Na2CO3 → Filtration → Ethanol precipitation	Drying of precipitate → Milling (optional bleaching/neutralization)	(Jayasinghe et al., 2022)

In addition to conventional extraction, several modern techniques, such as microwave-assisted, ultrasound-assisted, enzyme-assisted, and extrusion-based methods, have been developed and are often referred to as “green extraction” technologies. These approaches are typically integrated with traditional methods to enhance extraction efficiency, reduce processing time, minimize energy consumption, and lower environmental impact (Rahman et al., 2024; Savić Gajić et al., 2024; Ye et al., 2024; Ashour et al., 2025). Green extraction technologies offer sustainable alternatives to conventional methods by improving extraction efficiency while reducing solvent use, energy consumption, and processing time (Veršić Bratinčević et al., 2023).

Traditional techniques such as maceration and Soxhlet extraction often require large amounts of organic solvents, extended extraction periods, and high temperatures, which can degrade heat-sensitive bioactive compounds and lower extract quality. In contrast, green methods such as microwave-assisted extraction (MAE) and Ultrasound-Assisted Extraction (UAE) enhance extraction through rapid cell disruption and improved mass transfer, leading to higher yields and better preservation of valuable phytochemicals (Shrivastav et al., 2025).

The six principles of green extraction provide a comprehensive framework for sustainable process development, emphasizing renewable resources, safer solvents, reduced energy demand, by-product valorization, simplified processing, and production of high-quality biodegradable extracts (Undale et al., 2026).

However, despite their environmental and technical advantages, large-scale industrial implementation remains challenged by issues such as equipment cost, process standardization, and scale-up limitations. Overcoming these barriers requires pilot-scale optimization, continuous processing systems, hybrid technologies, and techno-economic and life cycle assessments to support sustainable commercialization (Fomo et al., 2020; Švarc-Gajić and Morais, 2022).

Due to its biocompatibility, non-antigenicity, and high chelating capacity, alginate has gained significant interest in biomedical and pharmaceutical uses (Batista et al., 2019). In drug formulations, it acts as a versatile excipient that stabilizes active compounds and controls their release profiles. Its carboxyl groups stay ionized at pH levels above 3–4, increasing solubility in higher pH environments and improving its ability to protect drugs and promote gastrointestinal absorption. These solubility and pH-responsive properties make alginate an ideal candidate for developing advanced, controlled-release drug delivery systems (Krishnamurthi et al., [[NoYear]]; Lomartire and Gonçalves, 2022).

Apart from pharmaceutical uses, alginate demonstrates exceptional viscosity-enhancing, gel-forming, and film-forming properties. Its water solubility, biodegradability, and chemical versatility support its broad application across multiple fields, including healthcare, food technology, catalysis, water purification, packaging, and cell immobilization (Sabalingam and Jayasuriya, 2019; Sharma and Bhende, 2024). In particular, alginate has gained prominence in tissue engineering and the fabrication of biomaterial scaffolds, where it provides structural support for cell growth and regeneration. Recent studies have further emphasized the functional attributes of alginic acid, such as elasticity, deformability, disintegration behavior, and compressibility, highlighting its expanding potential in innovative pharmaceutical formulation design (Chudasama et al., 2021).

#### 4.1.2 Biocompatibility of alginate

Alginate biocompatibility closely depends on its purity level and has been extensively tested both *in vivo* and *in vitro*. Alginate is a naturally occurring anionic polysaccharide mainly derived from brown seaweeds, and it is popular because of its excellent biocompatibility and its ability to form mild gels in the presence of divalent cations like Ca² (Wu et al., 2018). Alginate yield from brown seaweeds has been widely reported in the literature to vary considerably depending on the algal species and extraction conditions. Several studies have demonstrated that alginate yield typically falls within a moderate range relative to dry weight. For instance, alginate extracted from Sargassum latifolium was reported to yield approximately 17.5% (w/w) (Dalal et al., 2021). Under optimized extraction conditions, yields ranging from 17.47% to 24.08% (DW) have been documented, with higher values observed in species such as Sargassum cinereum (24.08 ± 0.33% DW), followed by Padina pavonica (21.13 ± 2.47% DW), *Dictyota dichotoma* (19.57 ± 3.60% DW), and *Turbinaria turbinata* (17.47 ± 0.26% DW) (El-Sheekh et al., 2024). The chemical makeup of alginate mainly influences its purity and, in turn, its biocompatibility. For example, alginates with a high amount of mannuronic acid (M) residues have been shown to cause greater immune responses and stimulate cytokine production up to ten times more than guluronic acid (G)-rich alginates (Ching et al., 2017). However, other research has observed minimal or no immune reactions around alginate implants, indicating that factors besides monomer composition might also play a role in the differing outcomes (Lei et al., 2017).

Variations in biocompatibility often result from impurities naturally found in seaweed-derived alginate. Impurities such as heavy metals, endotoxins, proteins, and polyphenolic compounds can elicit local inflammatory or foreign-body reactions at implantation or injection sites. Conversely, alginates produced by reputable commercial suppliers, which are usually subjected to thorough purification and quality control, seldom cause significant tissue damage or inflammatory responses (Lei et al., 2017). Supporting this, highly purified alginate obtained through multi-step extraction methods caused no notable foreign body reaction when implanted in animal models (Ayal et al., 2019). Subcutaneous injections of gels made from commercially purified alginate did not trigger significant inflammation in mice (Chan and Mooney, 2013). Using RAW 264.7 macrophage-like cells and bone marrow stromal cells (BMSCs), further findings showed that removing impurities not only reduces cytotoxicity and pro-inflammatory signaling but also improves the osteoinductive properties of alginate, highlighting the importance of purity in ensuring predictable biocompatibility (Torres ML. et al., 2019).

The biocompatibility of alginate–polycation microcapsules is also strongly influenced by the choice of polycation and its interaction with the alginate matrix. Substituting poly-L-lysine (PLL) with poly-L-ornithine (PLO) has been shown to decrease immune cell adhesion and improve microcapsule stability. Conversely, PLL-based membranes paired with certain alginate cores can provoke stronger immune responses, largely due to inadequate polycation penetration and poor membrane structural integrity. These observations suggest that the physicochemical interactions between alginate and polycations during membrane formation exert a more decisive effect on biocompatibility than the surface density of the polycation or the addition of an outer alginate coating (Tam et al., 2011).

#### 4.1.3 Biodegradability of alginate

In mammals, alginate is inherently non-degradable because they lack the alginase enzyme necessary to cleave its polymer chains. To render alginate degradation under physiological conditions, partial oxidation of the polymer is commonly employed (Jeon et al., 2010). Oxidized alginate becomes susceptible to degradation in aqueous environments, enabling its use as a biodegradable matrix for drug delivery and cell-based applications (Li et al., 2019).

Sodium periodate is widely used to oxidize alginate. Periodate oxidation cleaves the carbon–carbon bond within the cis-diol groups of uronate residues, converting the native chair conformation into an open-chain adduct that cannot maintain the original alginate backbone structure (Santos et al., 2018). This process typically leads to a modest reduction in molecular weight. Controlling the molecular weight distribution of alginate is therefore essential, as it allows decoupling of degradation kinetics from the mechanical properties of alginate hydrogels.

Partially oxidized alginates can form binary alginate gels composed of low- and high-molecular-weight fractions via ionic or covalent crosslinking, thereby providing tunable mechanical and degradation characteristics (Savarino et al., 2012).

#### 4.1.4 Factors affecting biodegradability of alginate

##### 4.1.4.1 Chemical modifications and blending

According to Barceló et al. (2022), the degradation rate of alginate-based biomaterials is heavily influenced by chemical modifications like the degree of oxidation. Greater oxidation speeds up hydrolytic chain scission, resulting in quicker biodegradation. Additionally, blending native alginate with oxidized variants allows precise control of degradation kinetics for targeted biomedical uses. The inclusion of gelatin also impacts the process by initially boosting viscosity and printability; however, its rapid release within 48 hours creates porosity in the network, further promoting structural breakdown.

##### 4.1.4.2 Structural features of alginate

Abka-Khajouei et al. (2022) emphasized that the structural composition of alginate plays a decisive role. The mannuronic/guluronic acid (M/G) ratio directly affects cross-linking strength, where G-rich alginates form stronger ionic networks, resulting in slower degradation. Conversely, M-rich alginates are more prone to enzymatic and hydrolytic cleavage. Molecular weight and degree of polymerization also determine degradation kinetics, with lower-molecular-weight alginates degrading more rapidly. Additionally, natural variations such as algal species, anatomical origin, and harvesting season influence polymer composition and subsequent biodegradability (Abka-Khajouei et al., 2022).

##### 4.1.4.3 Cross-linking density and environmental conditions

Łabowska et al. (2023) demonstrated that cross-linking conditions and storage environment significantly affect alginate hydrogel stability. Hydrogels with higher calcium chloride concentrations showed less shrinkage and slower water loss, while weakly cross-linked structures became brittle and deformed more rapidly. Additionally, environmental factors such as low temperature (7°C) and high humidity (95%) were found to maintain hydrogel dimensions and mechanical strength, emphasizing the importance of storage and processing conditions (Łabowska et al., 2023).

##### 4.1.4.4 Enzymatic degradation by alginate lyases

Li et al. (2023) reported that enzymatic degradation can be markedly accelerated by novel alginate lyases. For example, AlyRm3, a thermostable alginate lyase from *Rhodothermus marinus*, demonstrated exceptional catalytic activity (>37,000 U/mg) at elevated temperatures (70–90 °C). This enzyme efficiently cleaved alginate into disaccharides and trisaccharides, maintaining stability under harsh industrial conditions. Such findings illustrate the potential of engineered alginate lyases to regulate biodegradation in biomedical scaffolds while enabling industrial applications such as biofuel production and oligosaccharide generation (Li et al., 2023).

### 4.2 Fucoidan

Fucoidan is a sulfated polysaccharide predominantly derived from brown seaweed and represents one of their major fucose-rich polysaccharide constituents (Ale et al., 2011b). Brown macroalgae are generally rich in indigestible polysaccharides and serve as an important source of soluble dietary fibers, among which fucoidan is considered particularly significant (Ale et al., 2011b; Lim and Aida, 2017). Due to its structural complexity and wide range of biological activities, fucoidan has been studied for its medicinal potential. Fucoidan inhibits the growth of cancer cells by increasing apoptosis, causing cell cycle arrest, and preventing angiogenesis and metastasis, according to a number of experimental studies (Arokiarajan et al., 2022; George and Shrivastav, 2023).

In brown seaweeds, fucoidan is localized mainly within the cell walls, where it can account for approximately 5–20% of the algal dry weight. Fucoidan yield is markedly affected by factors such as the species of brown algae, environmental and seasonal conditions, as well as the extraction technique employed. Previous studies have demonstrated significant variability in yield, with higher values reported for *Ascophyllum nodosum* (23.41%) and *Fucus evanescens* (18.2%) using enzyme-assisted extraction (Nguyen et al., 2020; Maribu, 2021). In comparison, moderate yields were obtained from *Fucus virsoides* (13.19%) via microwave-assisted extraction (Dobrinčić et al., 2021), while lower yields were observed in *Sargassum muticum* (4.60%) using ultrasound-assisted methods (Flórez-Fernández et al., 2017). This variation underscores the combined influence of algal source and extraction strategy, where enzyme-assisted techniques often enhance yield through efficient disruption of cell walls and improved release of sulfated polysaccharides.

It is a water-soluble, highly branched matrix polysaccharide composed largely of L-fucose residues linked through α-(1→3) and/or alternating α-(1→4) glycosidic bonds, and contains a substantial proportion of sulfate esters, typically around 40%. Its molecular weight can reach up to 1,000,000 g/mol, and variations in molecular size, sulfation degree, branching, and overall structural arrangement largely determine its diverse pharmacological activities (Saeed et al., 2021; Chadwick et al., 2025).

#### 4.2.1 Extraction method of fucoidan polysaccharide

Various extraction strategies have been developed for isolating fucoidan, each offering distinct advantages and limitations. Microwave-assisted extraction (MAE) has demonstrated strong potential for the rapid recovery of sulfated polysaccharides. Rodriguez-Jasso et al. (2011) reported efficient fucoidan extraction using MAE, with alginate precipitation facilitating partial purification. This technique reduces solvent consumption and enables dual valorization of algal biomass; however, the resulting alginate typically remains crude, and MAE requires specialized equipment and precise energy control to prevent polysaccharide degradation. These factors have constrained their scalability for industrial use (Rodriguez-Jasso et al., 2011).

Ultrasound-Assisted Extraction (USAE) provides a gentler approach that shortens extraction time and preserves fucoidan’s structural integrity. Flórez-Fernández et al. (2017) successfully applied USAE to *Sargassum muticum*, achieving higher yields while minimizing thermal degradation through subsequent ethanol precipitation and dialysis. Hmelkov et al. (2018) enhanced this further by combining USAE with ion-exchange chromatography for *Fucus evanescens*, yielding relatively pure fucoidan with preserved molecular features. Despite these benefits, USAE faces challenges in large-scale implementation due to equipment limitations and the risk of partial depolymerization when sonication parameters are not carefully optimized.

Enzymatic extraction has gained considerable attention as an approach that maximizes structural preservation and enhances purity. Sanjeewa et al. (2017) demonstrated the effectiveness of food-grade enzymes in extracting fucoidan from *Sargassum horneri*, while Rhein-Knudsen et al. (2023) combined cellulases and alginate lyases to obtain high-purity fucoidan with elevated fucose and sulfate content and minimal molecular degradation. Although enzymatic methods deliver reproducible and structurally intact products, they involve multiple processing steps, require precise enzyme optimization, and incur higher operational costs.

Conventional acid and hot-water extractions remain widely used due to their simplicity. Usov et al. (2022) and Abu-Resha et al. (2025) reported effective recovery of highly sulfated fucoidan fractions using dilute acid, whereas El-Sheekh et al. (2025b) optimized hot-water extraction to obtain high yields with minimal loss of structural integrity. However, these techniques are often energy- and time-intensive and may produce variable results due to seasonal or environmental fluctuations in algal composition.

Overall, enzymatic extraction combined with selective purification appears to be the most reliable method for producing high-purity, biologically active fucoidan, striking the best balance between yield, structural preservation, and functional bioactivity. Physical extraction techniques such as MAE and USAE provide speed and solvent efficiency but face scalability constraints, while conventional chemical and hot-water extractions offer operational simplicity at the cost of reduced reproducibility and purity.

#### 4.2.2 Characterization techniques for seaweed-derived polysaccharides: from conventional methods to advanced analytical approaches

The structural and functional characterization of seaweed-derived polysaccharides is performed after the extraction, purification, and isolation of algal polysaccharides. Conventional methods, including colorimetric assays and basic chromatographic techniques, provide preliminary information on total carbohydrate content and monosaccharide composition, functional group identification by FTIR (Fourier transform infrared spectroscopy), and structure elucidation by NMR (nuclear magnetic resonance) spectroscopy.

Extraction temperature below 160 °C caused moisture volatilization and resulted in yield loss during ulvan extraction from *Ulva lactuca*. To analyze for the presence of sulphate content, a turbidimetric assay is performed. This colorimetric analysis combined with other spectroscopic methods (FTIR, Raman scattering, NMR) provides essential information on the structure of extracted algal polysaccharides (Roy et al., 2024; Fanina et al., 2026).

Recent advancements have introduced more sophisticated techniques, such as Fourier-transform infrared spectroscopy (Fourier-transform infrared spectroscopy), nuclear magnetic resonance (Nuclear magnetic resonance spectroscopy), and mass spectrometry (Mass spectrometry), which enable detailed structural elucidation. These techniques allow for the identification of functional groups, sulfation patterns, glycosidic linkages, and molecular conformations. Additionally, chromatographic methods such as high-performance liquid chromatography (High-performance liquid chromatography) and gel permeation chromatography (Gel permeation chromatography) are widely used to determine molecular weight distribution and monosaccharide composition (The Fourier transform in analytical science, 2024).

Another critical parameter for structural analysis is molecular weight determination. This analysis is done by size exclusion chromatography (SEC) equipped with a refractive index or UV-detector. SEC can measure the molecular weight of all algal polysaccharides as well as particle size distribution. For monomer composition analysis, several chromatographic techniques such as liquid chromatography (LC), high-performance liquid chromatography (HPLC), gas chromatography (GC) equipped with refractive index, and mass spectrometer detection are used. GC is used to characterize volatile compounds (such as alditol) in polysaccharides, such as in ulvan (Liu et al., 2023; Krishnan et al., 2024).

Complete structural analysis requires Fourier transform infrared spectroscopy (FTIR) coupled with nuclear magnetic resonance (NMR) spectroscopy. FTIR spectroscopy is performed within the 500–4000 cm−1 absorption band. Some characteristic bands for algal polysaccharides derived from seaweeds are 3200–3400 cm−1 representing O–H vibrations, 2927–2891 cm−1 corresponding to C–H stretching, 1638–1597 cm−1 representing C[double bond, length as m-dash]O stretching, a 1247–1257 cm−1 absorption band suggesting S[double bond, length as m-dash]O bond vibration and 1134–1150 cm−1 representing the stretching vibration of C–H bonds. Compared to traditional methods, these advanced analytical tools provide higher sensitivity, accuracy, and structural resolution, enabling a deeper understanding of structure–activity relationships in polysaccharides (Krishnan et al., 2024).

In future perspectives, metabolomics approaches, particularly those based on high-resolution mass spectrometry and nuclear magnetic resonance, offer promising opportunities for comprehensive profiling of seaweed-derived bioactive compounds. The integration of metabolomics can facilitate the identification of novel metabolites, improve quality control, and enhance our understanding of the interactions between polysaccharides and biological systems. Therefore, combining advanced characterization techniques with metabolomics is expected to significantly advance the development and application of fucoidan-based biomaterials (Adarshan et al., 2024).

#### 4.2.3 Structure of fucoidan

Fucoidans, also referred to as fucoidan-containing sulfated polysaccharides (FCSPs), are structurally complex, sulfated heteropolysaccharides primarily composed of L-fucose residues, most commonly linked through α-(1→3) and/or α-(1→4) glycosidic bonds (Zvyagintseva et al., 1999; Jayawardena et al., 2022). Their architecture is highly heterogeneous, frequently incorporating various additional monosaccharides such as galactose, mannose, xylose, glucose, and glucuronic acid, as well as uronic acids and O-acetyl substituents (Zvyagintseva et al., 1999). Sulfate groups typically occupy the C-2 and/or C-4 positions of the fucopyranosyl residues, with occasional sulfation at C-3 (Ale et al., 2011b).

Fucoidan molecular weight varies extensively, ranging from approximately 100 to 1600 kDa, depending on the algal species and the extraction protocols employed (Zhang and Row, 2015). Considerable structural diversity has been documented across different orders of brown algae. For instance, fucoidans classified in species within the order Fucales (such as *Fucus vesiculosus*, *Ascophyllum nodosum*, *F. serratus*) typically exhibit backbones composed of alternating α-(1→3) and α-(1→4) fucose linkages, accompanied by variable sulfation patterns (Dubois et al., 1951; Mabeau et al., 1990; Colliec et al., 1991; Patankar et al., 1993; Chevolot et al., 1999; Bilan et al., 2006). By contrast, Sargassum species synthesize galactofucans, characterized by a fucose-rich core branched with galactose, glucose, xylose, and uronic acids (Duarte et al., 2001). In Laminariales (e.g., *Laminaria japonica*, *Saccharina cichorioides*), fucoidans exhibit mixed α-(1→3), α-(1→4), and α-(1→2) linkages, with sulfation commonly occurring at C-2 and C-4 (Wang et al., 2010a; Vishchuk et al., 2013; Usoltseva et al., 2019). Unique structural patterns have also been reported, for example, the fucoidan from *Cladosiphon okamuranus* features a predominantly linear α-(1→3)-fucopyranose backbone with C-4 sulfation and occasional O-acetylation (Nagaoka et al., 1999).

Importantly, this extensive structural variability is influenced not only by algal taxonomy but also by differences in extraction and purification methods, which contributes to the complexity and occasional inconsistency in fucoidan classification across studies. Comprehensive structural elucidation typically requires a combination of analytical approaches, including methylation analysis, desulfation, chromatographic techniques, FTIR, NMR spectroscopy, and mass spectrometry (Jayawardena et al., 2022).

#### 4.2.4 Biocompatibility of fucoidan-based nanocarriers

Fucoidan, a sulfated polysaccharide predominantly obtained from brown seaweeds, has recently attracted substantial interest as a functional component in nanocarrier design due to its intrinsic bioactivity and excellent safety profile. As a naturally occurring marine polysaccharide, fucoidan exhibits high biocompatibility, characterized by low toxicity and minimal immunogenicity, making it a promising candidate for a wide range of biomedical applications (Alekseyenko et al., 2007; Fitton, 2011; Wu et al., 2016). Extensive *in vitro* and *in vivo* investigations have consistently demonstrated their favorable tolerability in mammalian systems, further reinforcing their clinical potential.

Beyond its passive biocompatibility, fucoidan possesses diverse pharmacological activities, including anticoagulant, antiviral, antioxidant, anti-inflammatory, and, notably, antitumor effects. It can induce apoptosis and cell-cycle arrest in various cancer cell lines, such as those derived from colon and breast tumors, while simultaneously modulating immune responses to promote antitumor immunity (Alekseyenko et al., 2007; Fitton, 2011; Atashrazm et al., 2015; Wu et al., 2016). These combined biological activities allow fucoidan-based nanocarriers to function not only as delivery platforms but also as intrinsically therapeutic agents.

Kimura et al. (2013) reported that fucoidan nanoparticles exhibit enhanced anticancer activity against osteosarcoma while maintaining excellent biocompatibility. Compared with native fucoidan, the nanoparticle formulation demonstrated superior pro-apoptotic effects, improved cellular permeability in Caco-2 models, and greater bioavailability, underscoring its promise as a safe and effective therapeutic candidate (Kimura et al., 2013).

Consistent with these findings, Chiang et al. (2021) demonstrated that fucoidan-based nanoparticles (FuNPs) achieved efficient cellular uptake, potent antitumor and antimetastatic effects, and an expanded therapeutic window in a breast cancer mouse model. These results further confirm the strong safety profile and therapeutic potential of FuNPs as inherently active nanomedicine formulations (Chiang et al., 2021).

In agreement with this evidence, Choi et al. (2025) developed fucoidan–chitosan (F/CS) nanocarriers loaded with TMB, Prussian blue, and glucose oxidase (F/CS@TPGOx) that combined chemodynamic, photothermal, and starvation therapies within the tumor microenvironment. Remarkably, at a concentration of only 4 μg/mL, F/CS@TPGOx reduced cancer cell viability to below 20% after four hours of incubation. Both *in vitro* and *in vivo* experiments confirmed their high therapeutic efficacy with minimal off-target toxicity, further highlighting the strong biocompatibility of fucoidan-based nanocarriers for clinical cancer therapy (Choi et al., 2025).

Beyond oncology (Liu M. et al., 2022), demonstrated the therapeutic versatility of fucoidan–chitosan nanoparticles (CFNs) in cardiovascular medicine. CFNs exhibited potent antioxidants and anti-inflammatory properties through reactive oxygen species (ROS) scavenging and modulation of inflammatory mediators. In addition, their ability to bind P-selectin on activated endothelial cells and platelets facilitated selective accumulation within atherosclerotic plaques, where they effectively reduced local oxidative stress, suppressed leukocyte recruitment, and prevented disease progression. These findings support the utility of CFNs as biocompatible and multifunctional platforms for cardiovascular nanomedicine (Liu M. et al., 2022).

In immunological applications, Yang et al. (2023) isolated fucoidan (S3) from *Sargassum thunbergii* and demonstrated its immuno-enhancing effects both *in vitro* and *in vivo*. In RAW 264.7 macrophages, S3 stimulated nitric oxide production, enhanced phagocytic activity, and upregulated TNF-α, IL-6, IL-1β, and IL-10 via NF-κB activation (Yang et al., 2023). Similarly, in zebrafish models, S3 promoted innate immune responses through enhanced NO release and NF-κB pathway activation. These results highlight its favorable biocompatibility and potential application as a safe bioactive ingredient in functional foods and nutritional supplements.

Furthermore, Atya et al. (2021) revealed potent antioxidant, anti-inflammatory, and hepatoprotective activities of fucoidans isolated from *Colpomenia sinuosa* (FCS) and *Sargassum prismaticum* (FSP). FCS exhibited stronger antioxidants and cytotoxic effects against hepatic cancer cells compared with FSP. *In vivo*, FCS significantly improved liver function markers, reduced oxidative stress and inflammatory mediators, and alleviated histopathological damage in a rat model of paracetamol-induced hepatotoxicity. These findings further underscore the strong biocompatibility and therapeutic versatility of brown algae–derived fucoidans, particularly FCS, in liver protection (Atya et al., 2021).

#### 4.2.5 Biodegradability of fucoidan-based nanocarriers

From a biodegradability standpoint, fucoidan’s polysaccharide backbone is naturally prone to enzymatic and metabolic breakdown within the body, enabling efficient clearance without long-term tissue accumulation. The resulting degradation products are non-toxic and can be readily metabolized or excreted, making fucoidan well-suited for use in sustained and controlled drug release systems. In comparison with other commonly used polysaccharides, such as chitosan and alginate, fucoidan offers the combined advantages of inherent biodegradability and intrinsic bioactivity. This unique dual functionality enhances its value in the design of layer-by-layer (LbL) nanostructures, where it contributes not only to structural integrity but also to therapeutic efficacy (Fitton, 2011; Wu et al., 2016).

#### 4.2.6 Factors affecting biodegradability

##### 4.2.6.1 Molecular weight

Low Mwt fucoidan experiences rapid hydrolytic and enzymatic degradation, but high Mwt fucoidan offers slower breakdown and greater structural stability.

##### 4.2.6.2 Degree of sulfation

A higher sulfation degree improves hydrophilicity and could enhance enzymatic recognition, but it can also slow degradation because of the strongest electrostatic interactions within the polymer network.

##### 4.2.6.3 pH sensitivity

Fucoidan destroys more quickly in acidic environments, for instance those found in tumor microenvironments or lysosomes, making it beneficial for pH-responsive and targeted drug release applications.

##### 4.2.6.4 Crosslinking density

Increased crosslinking density decreases degradation rates, allowing sustained structural integrity and increased drug release in long-term delivery systems.

## 5 Study of how structure influences material performance and bioactivity

### 5.1 Fucoidan

The biological activity and material performance of fucoidan are deeply affected by its molecular structure. L-fucose, the dominant monosaccharide in fucoidan, plays a central role in mediating its anticoagulant, antioxidant, and anti-inflammatory features, whereas additional sugars like mannose, glucose, and galactose strongly affect physicochemical properties, including molecular weight (Mwt), solubility, and interactions with biological targets (Hans et al., 2021; Mensah et al., 2023; Abdel-Tawwab et al., 2024). The degree and distribution of sulfate groups on fucose and other sugar residues are very critical because sulfation improves anti-inflammatory, antiviral, and anticoagulant activities (Ale et al., 2011b).

Mwt is another key structural determinant: high Mwt fucoidans offer extensive surfaces for biomolecular interactions, whereas low Mwt fractions may provide superior diffusion, cellular uptake, and antioxidant efficiency (Bilan et al., 2006). Branching patterns and three-dimensional conformations also modulate material characteristics and biological interactions such as mechanical strength, gelation, and film formation, factors essential for biomedical applications, including drug delivery, wound healing, and tissue scaffolding (Kusaykin et al., 2008).

Overall, the interplay between sulfation patterns, monosaccharide content, molecular weight, and supramolecular configuration controls the full spectrum of fucoidan’s biological activity and material performance. This determines the significance of comprehensive structural characterization to optimize its therapeutic applications.

### 5.2 Alginate and alginate-based composite materials

The biological properties of alginate-containing biomaterials are strongly structure-dependent (Sharma and Kishen, 2024). Key structural parameters, surface chemistry, including morphology, porosity, and particle size, directly affect drug loading efficiency, antibacterial behavior, release kinetics, and bio-mineralization potential. In composite microspheres, particularly those fabricated from bioglass and biopolymers, careful structural design is important for achieving desired biological outcomes.

### 5.3 BIOGLASS/SA–PVP microspheres

BIOGLASS/SA–PVP and drug-loaded BIOGLASS/SA–PVP microspheres have been effectively synthesized using ion crosslinking procedures (Bakr et al., 2025). XRD and FTIR techniques determined the formation of apatite, evidenced by characteristic phosphate vibration peaks at 605 and 565 cm^−1^, confirming improved mineralization potential. SEM micrographs revealed uniformly, spherical shaped microspheres, illustrating successful structural stability and fabrication. Dielectric measurements across 4 Hz–8 MHz also reinforced their physicochemical strength.

Significantly, the structural integrity and optimized surface properties of these microspheres enabled potent antibacterial effect against both Gram-negative and Gram-positive microorganisms, as shown in agar diffusion analyses. Additionally, hydroxyapatite was deposited on the microsphere surface after immersion in simulated bodily fluid (SBF), confirming their significant bioactivity and suitability for both drug delivery and bone tissue engineering applications (Bi et al., 2019).

### 5.4 BCA composite scaffolds (bioglass–chitosan–alginate)

The bioactivity of BCA scaffolds is strongly determined by their three-dimensional structure. Their interconnected porous building supports proliferation, cell adhesion, and migration, key processes for active bone regeneration. Optimal porosity (82%–87%) and pore size (140–200 μm) encourage nutrient and oxygen diffusion, improving metabolic activity and tissue ingrowth.

Sodium alginate promotes crosslinking density with chitosan, enhancing pore walls and improving mechanical stability, therefore generating a favorable microenvironment for osteoblast adherence (Lin et al., 2015). The inclusion of bioglass also increases mineralization by releasing Si, Ca², and P ions, which encourages hydroxyapatite formation on the scaffold surface (Bellucci et al., 2016). This mineral layer mimics native bone extracellular matrix, increasing osteoconductivity and bone repair. Accordingly, scaffold porosity, pore-wall stability, crosslinking degree, and bioactive ion release synergistically determine the regenerative performance and bioactivity of BCA scaffolds (Shi et al., 2022).

### 5.5 Multi-scale porosity in MBG–PCL scaffolds

According to (Gómez-Cerezo et al., 2021), the multi-scale porous architecture of MBG–PCL scaffolds profoundly affect their biological activity. Macropores generated by 3D printing facilitate nutrient exchange, vascularization, and cell migration. Microporosity within printed struts rises surface area, improving osteoblast adhesion and protein adsorption. Mesopores contributed by mesoporous bioglass (MBG) act as reservoirs for Ca², Si, and P ions, enhancing osteogenic differentiation and accelerating hydroxyapatite nucleation (Gómez-Cerezo et al., 2021).

This hierarchical porosity results in early and homogeneous mineralization, closely approaching the structure and function of natural bone. Additionally, microporosity has been shown to enhance osteoblast metabolic activity and upregulate RUNX2 expression, highlighting the important role of scaffold architecture in regulating osteogenesis and improving bone regeneration results.

## 6 Types of fucoidan in drug delivery

### 6.1 Species-specific fucoidans

Fucoidans have been extracted from abundant brown algal species, such as *Ascophyllum nodosum* (Medcalf and Larsen, 1977), *Cladosiphon okamuranus* (Sakai et al., 2003), *Hizikia fusiforme* (Li et al., 2006), *Sargassum stenophyllum* (Duarte et al., 2001), *Lobophora variegata* (Medeiros et al., 2008), *Padina gymnospora* (Silva et al., 2005), and *Saccharina latissima* (Bilan et al., 2010), among others. These species-specific fucoidans vary markedly in monosaccharide content, branching patterns, and sulfation degrees, resulting in various pharmacological outlines (Zayed et al., 2020). Evidence from experimental studies highlights unique bioactivities related to specific sources. Fucoidan from *Laminaria japonica* has been shown to enhance physical performance and decrease fatigue (Chen et al., 2015). Undaria pinnatifida fucoidan (UPF) improves angiogenesis, mitochondrial biogenesis, and oxidative muscle fiber development, enhancing performance and muscle mass (Yang et al., 2024). Moreover, a combined formulation of UPF and *Fucus vesiculosus* fucoidan (FVF) improved muscle size and strength in trained and sedentary mice, underscoring the significance of species-dependent structural properties in modulating muscle physiology (McBean et al., 2021).

### 6.2 High-molecular-weight fucoidan extracts

High-molecular-weight (HMW) fucoidan is usually isolated as crude polysaccharide segments from brown algae such as *Fucus vesiculosus*. These macromolecular sulfated polysaccharides represent a wide range of biological activities, including anticoagulant, anti-inflammatory, antiviral, and angiogenesis-regulating properties (Pozharitskaya et al., 2020). Due to their highly sulfated structure and size, HMW fucoidans outline strong interactions with cell membrane receptors and plasma proteins, which contribute to their biological activity; however, their large molecular weight may limit systemic absorption and tissue penetration, making them predominantly suitable for topical formulations, localized drug delivery, and wound dressing applications (Haggag et al., 2023).

The extraction method strongly affects fucoidan’s molecular weight (Mwt) distribution. Hot-water extraction typically yields higher Mwt fractions (~57.7 kDa) along with minor low-Mwt components (~2.7 kDa). In contrast, acid-based extraction (e.g., HCl treatment) gives smaller fractions of almost 46.4 kDa and 3.4 kDa (Junaidi, 2013). These variations highlight the importance of extraction conditions in defining fucoidan’s physicochemical properties and its suitability for specific drug delivery systems.

### 6.3 Low-molecular-weight fucoidan

Fucoidan’s biological efficacy is strongly dependent on molecular weight. While HMW fucoidan often displays limited solubility and cellular uptake, low-molecular-weight fucoidan (LMWF) exhibits significantly improved bioavailability and improved biological activity (Choi and Kim, 2013). LMWF represents superior anticoagulant and antioxidant activities (Wang et al., 2010b), enhances osteoblast proliferation for bone regeneration (Changotade et al., 2008), and induces endothelial cell formation (Lake et al., 2006).

LMWF can be produced using acid hydrolysis, enzymatic degradation, or gamma irradiation. Acid hydrolysis, however, regularly leads to uncontrolled desulfation and depolymerization, which can diminish biological activity (Pomin et al., 2005). Enzymatic degradation by fucoidanases derived from marine fungi and bacteria offers more selective processing, although enzyme behavior remains dependent on specific structural motifs and is not universally effective (Wu et al., 2011).

### 6.4 Sulfated fucoidans

Fucoidans are naturally sulfated polysaccharides whose biological activities are directly influenced by their substitution patterns and sulfation degree. Studies on fucoidans from *Ecklonia cava*, *Ascophyllum nodosum*, and *Undaria pinnatifida* illustrate that the degree and position of sulfate groups strongly affect bioactivity (Athukorala et al., 2006). Structural analyses show predominant sulfation at the O-2 position, with lesser substitution at O-3 and occasional 2,3-O-disulfation, correlating significantly with anticoagulant potency (Bilan et al., 2023).

Chemically enhanced sulfation further amplifies anticoagulant activity by prolonging prothrombin time and protecting plasmin from the inhibition of α2-antiplasmin (Soeda et al., 1992; Soeda et al., 1993). The anticoagulant effects of fucoidan are closely tied to both sulfate density and molecular weight (Chevolot et al., 1999).

Although sulfated fucoidans also demonstrate notable antiviral and anti-inflammatory activities, these biological applications are discussed in subsequent sections (Costa et al., 2010).

### 6.5 Chemically modified fucoidan

Chemical modifications of fucoidan polysaccarides, such as carboxymethylation, acetylation, phosphorylation, and oversulfation, have been extensively explored to enhance stability, solubility, and bioactivity. Carboxymethylated fucoidan represents improved water solubility and heightened antioxidant capacity (Zhou and Li, 2024), whereas oversulfated fucoidan illustrates substantially stronger anticoagulant effects compared with native forms (Mourão, 2004). Acetylated fucoidan from *Cladosiphon okamuranus* has shown a strong therapeutic effect, including inhibiting the adhesion of *Helicobacter pylori* to gastric epithelial cells and decreasing chronic colitis in animal models (Shibata et al., 1999; Matsumoto et al., 2004).

These chemical derivatives are strongly utilized in regenerative medicine and nanotechnology (Iqbal et al., 2024). The rational design of fucoidan-based nanocarriers depends on properties such as molecular weight, solubility, thermal stability, pH responsiveness, and sulfation degree, all of which affects their behavior in drug delivery systems (Chollet et al., 2016; Citkowska et al., 2019).

## 7 Types of alginate in drug delivery

Alginate is a linear copolymer of β-D-mannuronic (M) and α-L-guluronic (G) acid residues. Its features in drug delivery, such as mechanical strength, gelation behavior, degradation rate, permeability, and mucoadhesion, are determined primarily by its source, monomer composition (M/G ratio), molecular weight, and degree of chemical modifications (Abka-Khajouei et al., 2022; Doniparthi et al., 2023).

### 7.1 Source-dependent alginate

Alginate is predominantly extracted from brown algae, including *Laminaria*, *Ascophyllum*, and *Macrocystis*, although it can also be produced by bacteria such as *Pseudomonas aeruginosa* and *Azotobacter vinelandii* (Rehm and Valla, 1997). Algal alginate ruins the most widely utilized form because of its low cost, abundance, and well-established safety profile (Gheorghita Puscaselu et al., 2020).

Bacterial alginate offers advantages such as a more tightly regulated biosynthetic process, allowing for better control over the M/G block structure and molecular weight distribution. These properties contribute to improved gelation features, pore structure, and drug release kinetics, enhancing its suitability for advanced delivery systems (Díaz-Barrera et al., 2014). Thus, source-dependent compositional differences play a significant role in refining alginate-based wound dressings and controlled-release platforms (Patne et al., 2025).

### 7.2 Molecular-weight-based alginate

The molecular weight (Mwt) of alginate strongly affects its physicochemical features and determines its performance in drug delivery systems. High Mwt alginate forms more viscous solutions and robust gels, this make it ideal for wound dressings and sustained release formulations because of its moisture retention and mechanical stability (Al-Roujayee et al., 2025).

Depolymerized alginate derivatives, extracted via controlled acid hydrolysis, enzymatic degradation, or gamma irradiation, have gained attention for their enhanced solubility and bioactivity. These low Mwt derivatives resemble promising immunomodulatory and anti-inflammatory activities related to cancer, infection, and neurodegenerative diseases (Xu et al., 2016).

Differences in molecular weight also affect cellular interactions: hydrogels with lower Mwt possess larger mesh sizes and higher diffusivity, improving nutrient transfer and cell infiltration, whereas higher Mwt alginate offers increased stability and slower drug release (Favre et al., 2001; Boontheekul et al., 2005). Mwt control techniques are therefore essential for tailoring alginate to specific biological applications (Rosiak et al., 2021).

### 7.3 Alginate types based on M/G ratio

The M/G ratio is one of the most significant parameters directing alginate’s behavior in biological applications. G-rich alginates exhibit strong affinity for Ca^2+^ and form rigid, mechanically stable gels with slow drug release profiles, properties well-suited for long-term delivery systems (Román-Guerrero et al., 2025; Tordi et al., 2025). In contrast, M-rich alginates give softer, more elastic hydrogels with higher water uptake, valuable for tissue engineering and formulations demanding faster release or degradation (Liang et al., 2025).

Recent findings demonstrate that the M/G ratio also modulates cell responses, including immune interactions and macrophage polarization. This behavior directly affects hydrogel porosity, swelling behavior, and biocompatibility in wound healing contexts (Wu et al., 2024).

### 7.4 Chemically modified alginate

Extensive research has explored chemical modification of alginate, such as sulfation, phosphorylation, amidation, and polymer grafting, to improve its performance in drug delivery.

Sulfated alginate hydrogels exhibit improved anti-inflammatory and anticoagulant properties, making them promising candidates for therapeutic matrices and wound healing (Huang et al., 2023). Phosphorylated alginic acid is able to form stable injectable calcium-based hydrogels with enhanced bioactivity and mineralization capacity, enhancing applications in bone tissue engineering (Kim et al., 2015). Amidated alginate shows improved mechanical stability, structural consistency, and controlled swelling, addressing numerous limitations of unmodified alginate (Taubner et al., 2017).

Moreover, grafting with synthetic polymers such as polyethylene glycol (PEG) or polycaprolactone (PCL) improves solubility, tunable degradation, and sustained-release behavior (Chandel et al., 2016; Bibire et al., 2025). Collectively, these modifications broaden alginate’s biological applicability, encouraging precise control over drug release kinetics, biocompatibility, and cellular responses across regenerative medicine and nanocarrier-based therapeutics.

## 8 Formulations of alginate and fucoidan

The versatility of alginate and fucoidan has supported their integration into a broad range of pharmaceutical and biomedical delivery platforms. Their complementary physicochemical and biological characteristics enable the development of multifunctional systems that combine biocompatibility, structural integrity, and therapeutic bioactivity. Representative formulations for each polymer are summarized below.

### 8.1 Alginate-based formulations

#### 8.1.1 Hydrogels

Alginate hydrogels represent the most extensively explored formulations because of their excellent biocompatibility, injectability, and ability to maintain a moist wound environment. Crosslinking with Ca^2+^ ions produce a stable three-dimensional (3D) network that can be further functionalized with therapeutic agents, growth factors, or nanoparticles to improve biological activities (Abasalizadeh et al., 2020; Zhang et al., 2020).

#### 8.1.2 Films and membranes

Alginate films, fabricated via solvent casting or layer-by-layer assembly. This creates flexible and breathable barriers appropriate for controlled drug diffusion. These films can be loaded with antimicrobials or growth factors, and the incorporation of metallic nanoparticles, such as silver, has demonstrated improved antibacterial activity and enhanced wound healing (Astaneh and Fereydouni, 2024).

#### 8.1.3 Nanofibers

Electrospun alginate nanofibers procedure ECM-like fibrous scaffolds, which encourage cell adhesion and tissue regeneration (Tripathi et al., 2025). Blended alginate nanofibers demonstrate improved mechanical features and increased bioactivity, encouraging both fibroblast attachment and epithelial repair (Jeong et al., 2010).

#### 8.1.4 Microspheres and beads

Ionotropically crosslinked alginate microspheres and beads have been largely employed as carriers for many therapeutic agents because of their capacity to capture hydrophilic molecules (e.g., peptides, proteins) as well as hydrophobic drugs (George and Abraham, 2006). Their release profiles are normally governed by diffusion, making them particularly suitable for mucosal and oral delivery applications (Anal and Stevens, 2005). Studies have shown that alginate microspheres can efficiently encapsulate proteins such as lysozyme and insulin, protecting them from degradation under gastric conditions while enabling targeted intestinal release. Hybrid alginate-based microparticles have also been developed to deliver hydrophobic molecules, such as ketoprofen and quercetin, through mucosal surfaces with sustained release and improved bioavailability (Hariyadi et al., 2012; Pawar and Edgar, 2012).

#### 8.1.5 Sponges and foams

Freeze-dried alginate sponges and foams show exceptional absorbency, which is useful for managing highly exudative wounds. Their porous architecture promotes oxygen permeability, nutrient transport, and cellular infiltration, encouraging tissue regeneration (Hu et al., 2024). These matrices can also be functionalized with therapeutic agents for rapid wound healing, as illustrated in multiple reports (Boateng et al., 2008).

#### 8.1.6 Injectable *in-situ* gels

Alginate can form injectable gels that undergo *in situ* crosslinking under physiological conditions. These systems offer minimally invasive platforms for localized drug delivery, wound repair, and tumor therapy (Abasalizadeh et al., 2020). Lee and Mooney (2012) provided a comprehensive overview of alginate applications injectable hydrogels in regenerative medicine and controlled therapeutic delivery (Lee and Mooney, 2012).

### 8.2 Fucoidan-based formulations

#### 8.2.1 Nanoparticles and nanogels

Fucoidan-based nanoparticles and nanogels have gained significant attention due to their biological functions, which are imparted by their sulfated polysaccharide structure (Iqbal et al., 2024). The dense distribution of sulfate groups enables strong interactions with selectins, cell adhesion receptors involved in inflammation, leukocyte trafficking, and cancer progression. Because selectins are often overexpressed in inflamed and tumor tissues, fucoidan nanoparticles can selectively accumulate at these pathogenic sites, enhancing targeted therapeutic delivery (Cumashi et al., 2007). Encapsulating hydrophobic drugs such as cisplatin or doxorubicin within fucoidan nanogels can significantly improve drug solubility, stability, and antitumor efficacy compared to free drug formulations (Nguyen et al., 2024). Additionally, fucoidan nanoparticles have been engineered for controlled release of anti-inflammatory agents, effectively reducing cytokine expression and oxidative stress *in both in vitro* and *in vivo* applications (Afrin S. et al., 2025).

#### 8.2.2 Microspheres

Fucoidan microspheres have been created for controlled delivery of antioxidant and anti-inflammatory agents. Their release patterns are usually diffusion-controlled, providing consistent and long-lasting therapeutic effects. These microspheres also show improved stability in gastrointestinal conditions, supporting their potential for oral, mucosal, and colon-specific drug delivery (Iliou et al., 2022). Several studies confirm fucoidan’s potential as a natural polymeric carrier for controlled release and drug encapsulation (Haggag et al., 2023).

#### 8.2.3 Hydrogels

Incorporating fucoidan into alginate hydrogels forms composite matrices which integrate mechanical stability with bioactivity. Fucoidan exhibits anti-inflammatory, antioxidant, and pro-angiogenic effects, thereby improving tissue regeneration. In animal models, fucoidan-enriched hydrogels were able to promote granulation tissue formation, vascularization, and rapid wound closure compared with alginate alone (Soeda et al., 2000; Ghahtan et al., 2025).

#### 8.2.4 Conjugates and complexes

Fucoidan has been chemically linked with bioactive molecules such as proteins, growth factors, and synthetic polymers to improve stability, control release, and boost therapeutic functions. For example, fibroblast growth factor-2 incorporated into fucoidan–chitosan multilayer assemblies demonstrated prolonged release and significantly enhanced bioactivity for wound healing applications (Benbow et al., 2020). These conjugates support targeted delivery and expand fucoidan’s use in tumor therapy, tissue engineering, and multifunctional therapeutic systems, combining antioxidant, anti-inflammatory, and anticancer properties within a single platform (Barbosa et al., 2019; Haggag et al., 2023).

#### 8.2.5 Films and coatings

Fucoidan-based surface coatings enhance hemocompatibility and promote endothelial cell attachment, making them appropriate for vascular grafts, implantable devices, and wound care materials. When applied alone or in combination with other polymers, these coatings generate bioactive interfaces that enhance healing outcomes (Yao et al., 2020).

#### 8.2.6 Liposomes and emulsions

Fucoidan-integrated liposomes were able to increase the stability, solubility, and bioavailability of poorly water-soluble compounds while preserving their therapeutic effects. These carriers improve sustained release and enable combination strategies via co-encapsulation of multiple drugs, broadening their utility in pharmaceutical and biomedical functions (Han et al., 2025).

## 9 Mechanisms of drug delivery

Fucoidan and alginate have complementary physicochemical and biological properties, making them well-suited for the development of advanced drug delivery platforms (Barbosa et al., 2019). Their contributions to therapeutic delivery can be illustrated through numerous key mechanisms. Environmental responsiveness, ionic interactions and polymer structure control these processes.

### 9.1 Ionotropic gelation

**Figure 2:**
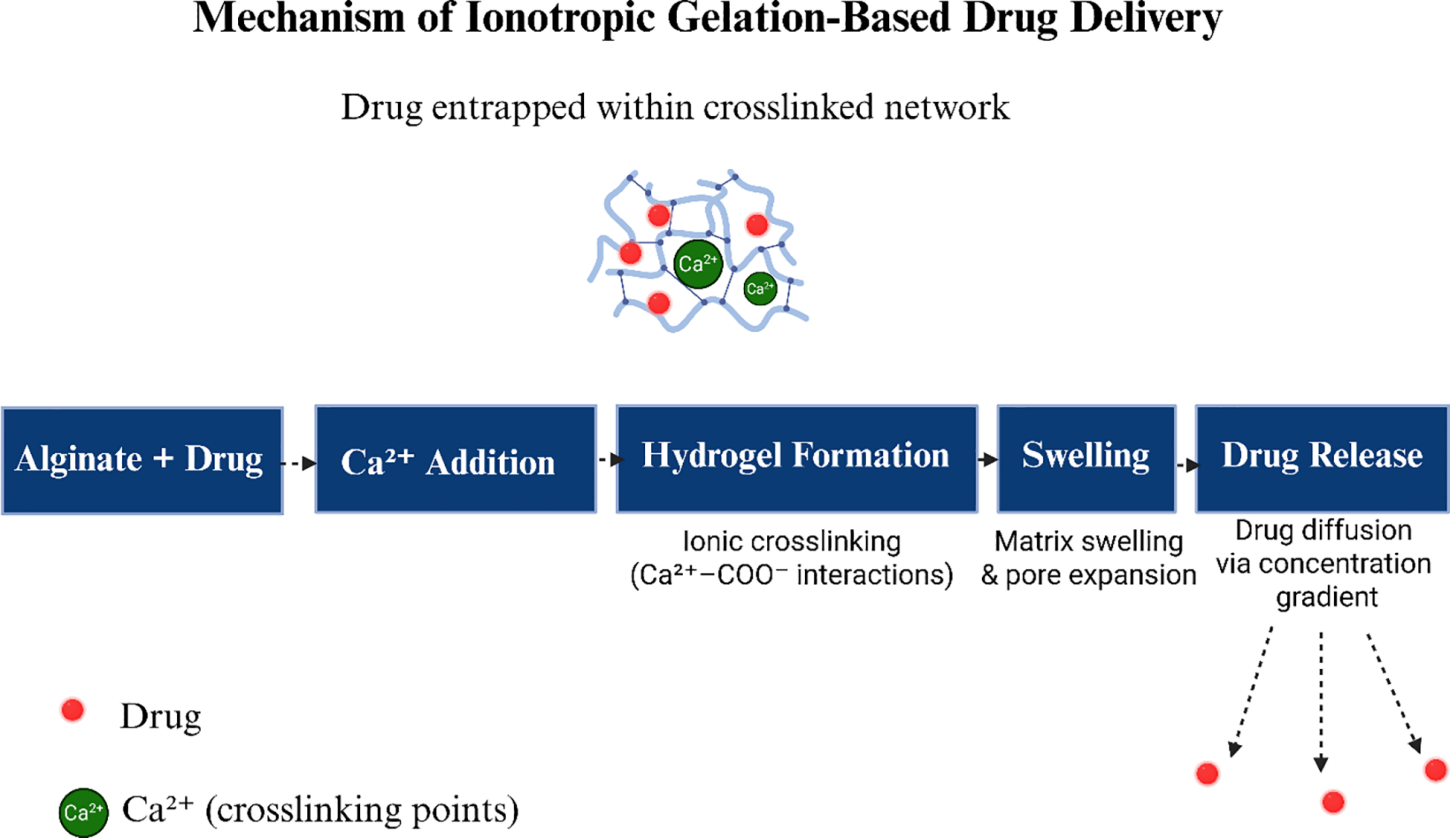
Illustration of ionotropic gelation-based drug delivery. Calcium ions crosslink alginate chains to form a hydrogel network that entraps drug molecules. The matrix then swells in aqueous conditions, allowing gradual drug diffusion release.

Alginate readily undergoes ionic crosslinking with divalent cations, most commonly calcium, resulting in robust three-dimensional hydrogel networks (Smith and Senior, 2021). This ionotropic gelation not only stabilizes the polymer matrix but also promotes efficient encapsulation of therapeutic agents, providing a foundation for controlled and sustained drug release (Iqbal et al., 2024). Also, Ca^2+^ ions preferentially bind with guluronic acid (G) blocks during the gelation process, creating connecting zones that gradually solidify the network through a cooperative binding mechanism. This interaction decreases electrostatic repulsion between polymer chains and facilitates their assembly into a three-dimensional structure. The distribution of space of these transition zones defines the hydrogel’s pore size and mechanical strength, which in turn affects drug entrapment efficiency and release behavior (Hu et al., 2021). The sequence of steps in mechanism, including ionic crosslinking, hydrogel formation and drug entrapment followed by release, is illustrated in **Figure 2**.

### 9.2 Diffusion-controlled release

Following encapsulation, drug release typically proceeds via diffusion-dominated mechanisms, where the kinetics are strongly influenced by factors including polymer crosslinking density, pore architecture, and the swelling behavior of the hydrogel (Alfinaikh et al., 2025). Water penetration into the hydrogel matrix, which causes polymer relaxation and expands the network’s free volume, controls diffusion at the molecular level. Drug molecules can move outward along a concentration gradient thanks to the interconnecting routes that this mechanism develops. Consequently, crosslinking density and network homogeneity have a major influence on the degree of mobility, substantially crosslinked systems limit molecular mobility and delay drug release (Leong et al., 2016). These systems can maintain extended and steady release profiles, thus decrease administration frequency and improve therapeutic agents (Zhang et al., 2022).

### 9.3 pH-responsive release

Both alginate and fucoidan have ionizable functional groups, making them sensitive to environmental pH (Barbosa et al., 2019). Under acidic conditions, alginate networks typically contract, limiting drug diffusion; at neutral or alkaline pH, they swell and enhance permeability. This pH-responsive behavior makes these polymers ideal for oral formulations and wound-healing applications where pH variation plays a key role (Meng et al., 2024). The ionization state of carboxyl groups throughout the polymer structure is responsible for the occurrence. This process increases electrostatic repulsion along chains at higher pH levels, which leads to increased swelling and network expansion. By contrast, protonation lowers charge density in acidic environments, which causes the matrix to compress and diffusion to be restricted. A vital component of localized medication delivery is this reversible structural alteration (Sun and Tan, 2013).

### 9.4 Mucoadhesion

One of the key advantages of both alginate and fucoidan is their strong mucoadhesive ability, which comes from hydrogen bonding and electrostatic interactions with mucin glycoproteins (Uzunalli, 2019; Smith and Senior, 2021). These interactions help extend the retention of drug carriers on mucosal surfaces, thereby boosting local absorption and overall therapeutic effectiveness. The mucoadhesive process generally takes two stages initial contact and wetting of the mucosal surface, then the interpenetration of polymer chains into the mucus layer. Secondary interactions such as hydrogen bonding and electrostatic attraction help to improve retention time and localized medication absorption (Yermak et al., 2022).

### 9.5 Stimuli-responsive systems

Both polysaccharides can be engineered into stimuli-responsive delivery systems in which drug release is modulated by external or internal triggers such as ionic strength, enzymatic action, or temperature fluctuations (Meng et al., 2024; Alfinaikh et al., 2025). For instance, in diabetic wound models, chitosan, fucoidan-alginate composites confirmed responsiveness to local microenvironmental cues, enabling time-dependent release of bioactive molecules, accelerating tissue regeneration (Uzunalli, 2019). These systems use structural shifts within the polymer network to respond to environmental influences. Modifications in ionic properties or enzyme performance can break crosslinking links or modify polymer structure, resulting in targeted matrix expansion or disintegration. This allows for specific control of drug delivery in response to certain biological states (Besiri et al., 2023).

### 9.6 Cell and growth factor delivery

In addition to small-molecule therapeutics, alginate–fucoidan matrices are increasingly applied for the delivery of cells and growth factors (Pallikkunnel et al., 2024). Their inherent biocompatibility and biological activity help protect encapsulated cells or proteins from degradation while promoting angiogenesis and tissue repair. This approach has shown considerable promise in wound healing and regenerative medicine, where controlled and sustained release of bioactive factors accelerates recovery (Aderibigbe and Buyana, 2018). The hydrogel matrix acts as a semi-permeable barrier, protecting capsule cells while enabling oxygen, nutrients, and transmitting chemicals to pass through. Cell proliferation and tissue regeneration are supported by the persistent release of growth factors, which is additionally promoted by gradual matrix disintegration or modification (Ghasemi et al., 2021).

## 10 Drug delivery system using alginate polysaccharide

Alginate-based biopolymeric nanoparticles have become highly promising carriers for active drug delivery because of their biodegradability, biocompatibility, and innate bioadhesive properties. Alginates are naturally occurring, water-soluble, and non-toxic polysaccharides composed of linear blocks of L-guluronic (G) and D-mannuronic (M) acids. The arrangement of these monomers greatly affects their biomedical effectiveness. They are particularly well suited for applications in wound healing, tissue regeneration, and controlled drug release due to their excellent gel-forming ability and structural similarity to the extracellular matrix. Recent studies highlight alginate’s versatility as a delivery vehicle across various administration methods, as well as its potential in composite and hybrid systems designed for advanced wound management and therapeutic modulation (Abourehab et al., 2022).

### 10.1 Oral drug delivery

Oral drug delivery is generally favored for its convenience, patient compliance, and non-invasive nature; however, its therapeutic activity is often limited by physiological barriers, including hydrolysis, enzymatic degradation, and poor permeability across the gastrointestinal epithelium (Barbosa et al., 2019).

To overcome these challenges, many coating strategies have been developed to enhance the stability and performance of CaCO_3_-based carriers for oral delivery. For example, hyaluronic acid–coated CaCO_3_ nanoparticles (HA/CCNPs) have been shown to protect insulin (INS) from acidic gastric degradation, retaining nearly 69% of the drug after 90 minutes in simulated gastric fluid, compared to only 9% retention in uncoated particles. These coated systems also maintained structural integrity, demonstrated efficient internalization into intestinal epithelial cells, and significantly lowered blood glucose levels in diabetic rats, outperforming subcutaneous insulin administration (Banks et al., 2019).

Similarly, alginate–chitosan-coated CaCO_3_ particles prepared through layer-by-layer assembly showed reduced burst release and improved intestinal uptake of encapsulated vaccines and therapeutic agents. Further improvements have been made by developing pH-responsive composite hydrogels, where alginate can be combined with agar, gum tragacanth, or chemically modified to enhance mechanical strength and control swelling behavior. These hydrogels offer strong protection in the acidic conditions of the stomach while allowing sustained and targeted release at neutral intestinal pH, making them suitable for oral delivery of vaccines, peptides, proteins, and small-molecule drugs with better bioavailability and stability (Sudareva et al., 2018; Liu et al., 2020; Huang et al., 2022).

### 10.2 Injectable and implantable systems

Implantable drug delivery systems provide various benefits, including targeted delivery of therapeutics at lower doses, fewer systemic side effects, and better patient compliance. Among the many polymers used in making implants, biopolymers, especially polysaccharides, are widely recognized for their ability to control and predictably modulate drug release profiles. Recent studies have concentrated on developing polysaccharide-based implants, supported by a thorough understanding of polymers like alginate (Salave et al., 2022).

Alginates are obtained from the cell walls of brown algae such as *Laminaria hyperborea*, *Ascophyllum nodosum*, and *Macrocystis pyrifera*. Due to their biocompatibility, gentle gelation behavior, and adjustable physicochemical properties, alginates have significant potential as biomaterials for controlled drug delivery. Alginates undergo hydrolytic cleavage in acidic environments through a clearly defined sequence of reactions: (a) the glycosidic oxygen atom is protonated; (b) the resulting conjugate is hydrolyzed to form a carboxonium ion and a non-reducing end; and (c) the carboxonium ion is quickly hydrated to produce a reducing end. Importantly, sodium alginate has good storage stability and can be kept dry and powdered at room temperature for several months without signs of deterioration (Sudareva et al., 2018; Salave et al., 2022).

### 10.3 Drug delivery system using fucoidan polysaccharide

Fucoidan exhibits a range of favorable biopharmaceutical properties, supporting its growing incorporation into pharmaceutical formulations to enhance therapeutic activity and disease control (Anisha et al., 2022; Haggag et al., 2023). Fucoidan has been widely studied as a functional biomaterial in drug delivery systems due to its polyanionic nature, excellent biocompatibility and capacity to interact with a wide range of medicinal agents. The existence of groups of sulfates allows for electrostatic interactions with positively charged polymers like chitosan, allowing the development of stable polyelectrolyte polymers capable of efficiently enveloping medicines and protecting them against premature destruction. These tools have shown enhanced regulated release behavior and stable drugs in physiological environments (Ale et al., 2011a; Barbosa et al., 2019). Fucoidan-based nanoparticles and nanocomposites have also demonstrated impressive outcomes in directed medication delivery. Particularly, fucoidan connects to selective proteins, specifically P-selectin, which is high in levels in inflamed and malignant tissues. This relationship reduces global adverse reactions while increasing the accumulation of pharmacological transporters at illness locations through active and passive selective mechanisms. Fucoidan-modified polymeric nanocarriers have been shown in numerous investigations to contribute to therapeutic effectiveness and cellular absorption of chemotherapy drugs (Fitton, 2011; Fernando et al., 2019). Fucoidan has been added to hydrogels, nanogels, and microspheres to provide long-term, targeted drug delivery. These technologies offer a three-dimensional network that is hydrated, allowing for extended-release characteristics and retaining stability of drugs (Farasati Far et al., 2022).

### 10.4 Mucoadhesive properties of fucoidan-based systems

Mucoadhesive formulations increase the time drugs stay on mucosal surfaces through polymer hydration, chain relaxation, and specific interactions with mucin proteins. Although fucoidan alone shows relatively weak mucoadhesive properties, combining it with complementary polymers, especially chitosan, greatly improves its ability to bind to mucus glycoproteins. Fucoidan–chitosan nanoparticles have demonstrated improved oral delivery of methotrexate, resulting in a seven-fold increase in antitumor activity compared to the free drug. Likewise, fucoidan–gelatin microspheres have shown better swelling ability and mucoadhesion, supporting their effectiveness for vaginal delivery of posaconazole. Overall, these findings emphasize fucoidan’s significant potential as a key component in mucoadhesive drug delivery systems (Szekalska et al., 2021; Haggag et al., 2023).

### 10.5 pH response

pH-responsive drug delivery is highly effective in targeting the acidic tumor microenvironment while reducing off-target effects in normal tissues. Due to the sulfonic acid groups of fucoidan, it exhibits inherent pH sensitivity; however, it is typically combined with complementary polymers like chitosan to optimize targeted release. These hybrid systems are often administered intravenously to prevent premature drug release in the gastrointestinal tract. Research has shown that fucoidan–chitosan nanoparticles can improve peptide delivery for autoimmune disorders, enable multi-stimuli–responsive doxorubicin (DOX) release for tumor therapy, and support sustained oral delivery of curcumin. Overall, fucoidan-based pH-sensitive carriers improve drug stability, control release kinetics, enhance absorption, and ultimately improve therapeutic outcomes (Lee and Huang, 2019).

### 10.6 Temperature response

Natural polysaccharides often show thermo-responsive behavior, either naturally or after chemical modification, usually through lower critical solution temperature (LCST) transitions, where a solution gels upon heating, or upper critical solution temperature (UCST) transitions, where a gel turns into a solution as temperature (Lee and Huang, 2019). Although fucoidan alone has limited gelling ability because of its low viscosity, its rheological properties are affected by factors such as algal species, molecular weight, and sulfate group content. When combined with complementary polymers like chitosan, gelatin, or β-glycerophosphate, fucoidan helps form hydrogels through hydrogen bonding and electrostatic interactions. These thermo-responsive hydrogels have strong potential in biomedical fields, especially in drug delivery and tissue engineering. For example, fucoidan–chitosan hydrogels loaded with VEGF and SDF-1 have been shown to promote vascularization and increase mesenchymal stem cell growth, with gelation happening at body temperature, enabling minimally invasive *in situ* administration (Graham et al., 2019; Zhang et al., 2021).

### 10.7 Enzymatic response

Fucoidan polysaccharide mainly consists of α-(1,3)-linked fucose residues, with smaller amounts of other linkages and monosaccharides. This structure makes fucoidan relatively resistant to breakdown by common digestive glycosidases. However, like other polysaccharides, it can be enzymatically cleaved by proteins such as matrix metalloproteinases (MMPs) and hyaluronidase, which are highly expressed in cancer microenvironments. Notably, fucoidan has also been reported to inhibit MMP overexpression, indicating a dual role in modulating harmful enzymatic activity while also serving as an enzyme-responsive biomaterial. Although this property remains underexplored, it presents a promising opportunity for developing site-specific drug delivery systems and highly sensitive diagnostic and theranostic platforms (Haggag et al., 2023).

### 10.8 Targeting ligand

**Figure 3:**
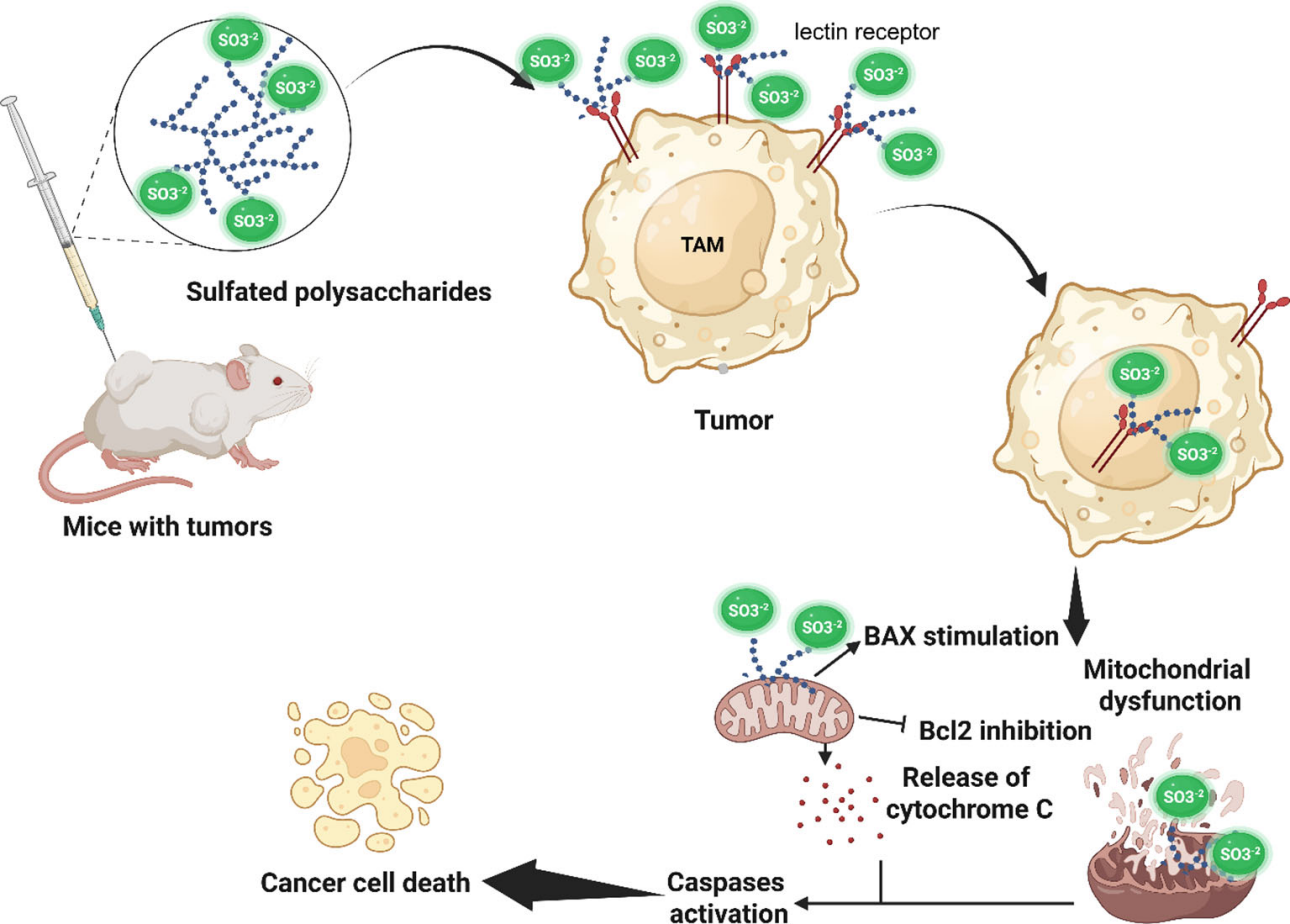
Mechanism of sulfated polysaccharide–induced apoptosis in tumor-associated macrophages leading to cancer cell death.

Ligand-mediated endocytosis can increase drug buildup in cancer cells while decreasing off-target toxicity in healthy tissues. Sulfated polysaccharides like fucoidan have a strong affinity for lectin receptors, especially p-selectin, which is overexpressed on activated platelets and many tumor cells and plays a key role in metastatic progression, as shown in **Figure 3**. Fucoidan-based nanoparticles demonstrate high binding affinity to both p-selectin and EGFR, enabling precise targeted drug delivery (Abd Elrahman and Mansour, 2019). Additionally, radiolabeled low-molecular-weight fucoidan has been successfully used for *in vivo* imaging of arterial thrombi and ischemic lesions due to its exceptional selectivity for p-selectin. Beyond cancer treatment, fucoidan has demonstrated therapeutic potential in pulmonary arterial hypertension, where it reduces hypoxia-induced vascular remodeling by targeting p-selectin overexpression (Novoyatleva et al., 2019; Liu X. et al., 2022).

## 11 Alginate and fucoidan applications

### 11.1 Alginate-based applications

**Figure 4:**
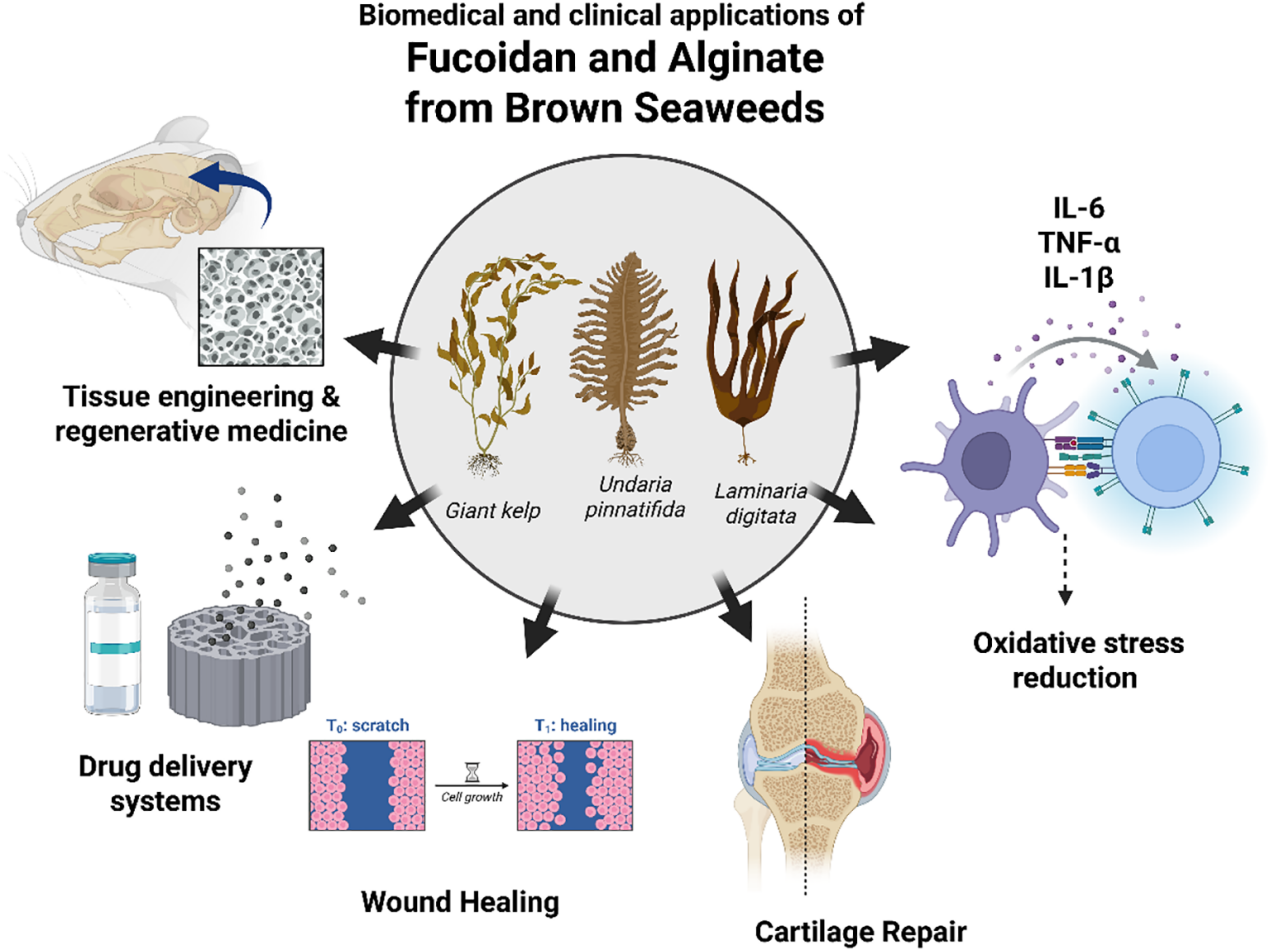
Biomedical applications of fucoidan and alginate derived from brown seaweeds: from scaffold fabrication to immune modulation and tissue repair.

Alginate dressings are widely used in clinical settings because they can form hydrophilic gels upon contact with wound exudate, creating a moist microenvironment that enhances autolytic debridement and speeds up tissue regeneration (Aderibigbe and Buyana, 2018). Their high absorbency allows for effective management of exudative wounds while maintaining an ideal balance between moisture retention and drainage, as shown in **Figure 4** (Sanghvi et al., 2025).

The intrinsic calcium ions in alginate confer a strong hemostatic effect, as ion exchange between calcium and sodium at the wound surface improves platelet aggregation and rapid clot formation, an advantage for bleeding control in acute injuries (Rahman et al., 2024).

Alginate-based matrices have also been developed as delivery systems for extended release of therapeutics. For example, alginate–gelatin microfibers integrated into transdermal patches enable the slow release of antibiotics like rifampicin, providing localized antimicrobial effects (Sharma et al., 2021). Alginate hydrogels and microspheres have also been utilized for the controlled delivery of growth factors and for encapsulating mesenchymal stem cells from adipose tissue and umbilical cord sources, promoting granulation tissue formation, neovascularization, and wound healing (Sheng et al., 2023).

The safety, biocompatibility, and biodegradability of alginate have reinforced the development of several commercial wound-care products. Dressings such as Kaltostat, Sorbsan, and Algisite are routinely applied for chronic burns, ulcers, donor sites, and post-surgical wounds (Agren, 1996; Duciel et al., 2025), illustrating the successful clinical translation of alginate-based technologies.

### 11.2 Fucoidan-based applications

Fucoidan provides additional therapeutic effects due to its multifunctional bioactivity as illustrated in **Figure 4**. As a sulfated polysaccharide, it exhibits potent anti-inflammatory features by suppressing the production of excessive cytokine and limiting chronic inflammatory responses common in non-healing wounds (Andryukov et al., 2020). Fucoidan also improves angiogenesis and promotes fibroblast proliferation, which facilitate granulation tissue development and collagen synthesis (Kim et al., 2018).

Its antibacterial and antioxidant activities further support wound healing by reducing reactive oxygen species and microbial burden in the wound bed (Lu et al., 2022). Beyond soft tissue repair, fucoidan can modulate extracellular matrix remodeling; for example, it inhibits leiomyoma cell proliferation and downregulates ECM-associated proteins, underscoring its regulatory effects on tissue restructuring (Chen et al., 2018).

Fucoidan has also been incorporated into composite scaffolds for tissue engineering applications. A notable example is the chitosan–alginate–fucoidan matrix developed by (Venkatesan et al., 2014), where the inclusion of fucoidan improved cell proliferation, protein synthesis, and mineral deposition compared with scaffolds which lack fucoidan. These findings highlight fucoidan’s role as a bioactive enhancer within natural polymer composites which expand its use in bone regeneration and other regenerative therapies (Venkatesan et al., 2014).

### 11.3 Applications for wound healing of alginate and fucoidan

**Figure 5:**
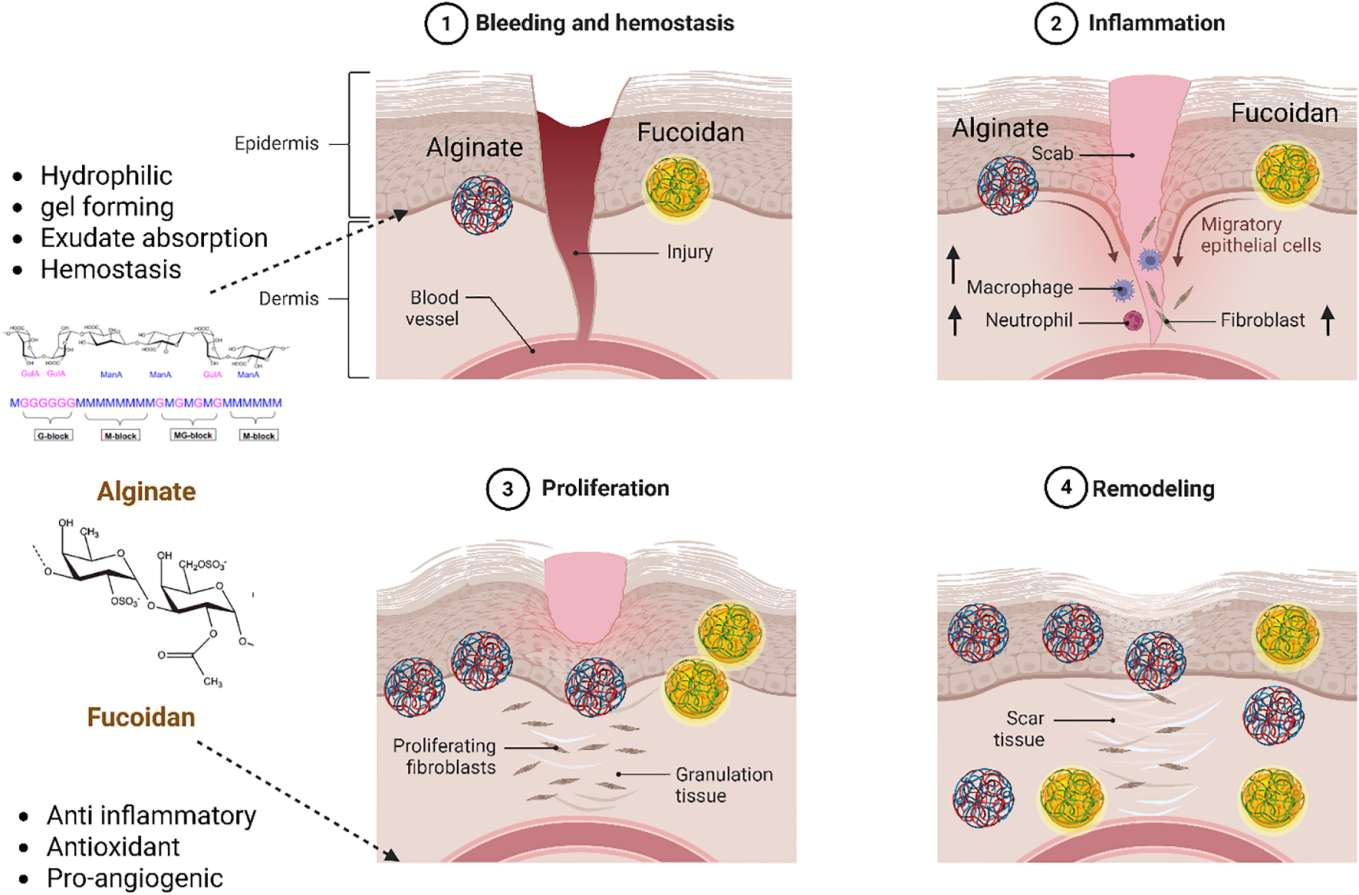
Synergistic effects of alginate and fucoidan across the four phases of wound healing.

Alginate and fucoidan possess biological features that make them suitable for recent wound treatment techniques, as shown in **Figure 5**. Alginate is a gel-forming matrix that absorbs excess bodily fluid, maintains a moist environment, remains hydrophilic, and promotes hemorrhage through calcium-mediated clot formation (Al-Roujayee et al., 2025). Fucoidan exhibits similar properties through its biological functions, including reducing oxidative stress, modulating inflammation, and stimulating angiogenesis (Abdollah et al., 2023). Together, these polysaccharides form multifunctional wound dressings that not only serve as physical protective barriers but also effectively promote tissue repair.

Preclinical studies demonstrate that fucoidan–alginate composites accelerate collagen deposition, epithelial remodeling, and promote fibroblast proliferation more effectively than each component alone (Rao et al., 2022). Extracellular matrix (ECM) modification and neovascularization are further enhanced by their ability to attract and stabilize growth regulators like VEGF and FGF (Vasuthas et al., 2025). Because of the unique properties of the fucoidan and alginate combination, which imparts distinctive characteristics to this compound, it is a potential biomaterial for treating both chronic and acute wounds.

## 12 *In vitro* and *in vivo* illustration

According to extensive *in vitro* research, alginate and fucoidan structures support fibroblast attachment and division as well as endothelial cell migration, both of which are crucial steps in wound repair (Vasuthas et al., 2025). It has been shown that fucoidan-containing hydrogels increase fibroblasts’ production of VEGF, thereby promoting angiogenesis and tissue regeneration (Unnikrishnan Meenakshi et al., 2024). As outlined in **Table 2**, *in vivo* studies, including diabetic and chronic wound models, further support these findings. Mixed alginate–fucoidan structures speed up wound contraction, promote re-epithelialization, and facilitate the growth of granulation tissue. The stimulation of key signaling pathways such as HIF-1α, Nrf2, and AKT enhances angiogenesis, reduces oxidative stress, and improves cell survival (Wen et al., 2023). ECM-like structures that encourage cell adhesion, proliferation, and controlled bioactive release are provided by innovative approaches such as electrospun nanofibers. In full-thickness wound models, gelatin/alginate-based skin replacements reinforced with nanofibers have shown significant improvements (Bal-Öztürk et al., 2024). Additionally, complex systems combining therapeutic nanoparticles with fucoidan or alginate have been developed. Silver nanoparticle-coated gelatin/fucoidan nanogels (Ag@Gel/Fu), which exhibit potent antibacterial activity, also boost angiogenesis and enhance fibroblast production *in vivo* (Hao et al., 2025). These multifunctional approaches illustrate how fucoidan and alginate can be integrated with nanostructures to offer next-generation wound treatments.

**Table 2:** Overview of fucoidan and alginate drug delivery systems: sources, types, applications, delivery modes, and evaluation parameters.

Algal source	Type of polysaccharide	Type of delivery system	Applications	Measured parameters (*in vitro*/*in vivo*)	Reference
(*Laminaria digitata*, *Macrocystis pyrifera*)	Alginate	Nanoparticles/Hydrogels	Controlled drug release, mucoadhesion	*In vitro*: release profile, mucoadhesion test; *In vivo*: stability, uptake efficiency	(Atmaca et al., 2022; Manna et al., 2025)
(*Fucus vesiculosus*)	Fucoidan	Nanoparticles/Hydrogels	Targeted drug delivery, anti-inflammatory	*In vitro*: cell viability (MTT), uptake (flow cytometry), cytokine assays (IL-6, TNF-α); *In vivo*: wound closure %, histology (H&E), angiogenesis markers (VEGF)	(Haggag et al., 2023; Sanghvi et al., 2025)
(*Sargassum muticum*)	Fucoidan + Alginate	Electrospun nanofibers	Wound healing, antimicrobial delivery	*In vitro*: antimicrobial assay (disk diffusion, MIC), fibroblast proliferation; *In vivo*: wound contraction %, collagen deposition, bacterial load reduction	(Alazaiza et al., 2023; Sanghvi et al., 2025)
(*Undaria pinnatifida*)	Fucoidan	Hydrogel/Dressing	Anti-inflammatory, angiogenic wound healing	*In vitro*: fibroblast proliferation, cytokine modulation (ELISA); *In vivo*: re-epithelialization, angiogenesis (VEGF), histology	(Kuznetsova et al., 2020)
(*Ascophyllum nodosum*)	Alginate	Hydrogel/Dressing	Moisture retention, exudate absorption	*In vitro*: swelling and water uptake; *In vivo*: wound moisture balance, pain scoring, autolytic debridement	(Aderibigbe and Buyana, 2018)
(*Laminaria japonica*)	Alginate	Injectable *in-situ* gel (doxorubicin-loaded)	Localized cancer therapy	*In vitro*: drug release, cytotoxicity (MTT); *In vivo*: tumor regression, survival analysis	(Vikas et al., 2023; Velapure et al., 2025)
(*Fucus vesiculosus*)	Fucoidan + Alginate	Hydrogel Dressing (clinical trial)	Diabetic and chronic wound repair.	*Clinical*: granulation tissue scoring, pain reduction, re-epithelialization rate.	(Haggag et al., 2023)

## 13 *In vitro* experimentation

More *in vitro* studies have shown that combining alginate and fucoidan provides biological benefits that extend beyond improved material qualities. Fucoidan’s sulfated structure, which matches glycosaminoglycans and improves matrix to cell connections, is also responsible for these effects in addition to their physicochemical affinity (Barbosa et al., 2019). For example, collagen and fucoidan mix films increased fibroblast migration and growth without causing cytotoxicity (Perumal et al., 2018). In immune cell models stimulated with lipopolysaccharide (LPS), fucoidan also demonstrated strong immunomodulatory properties, significantly reducing the production of anti-inflammatory cytokines like tumor necrosis factor-α (TNF-α), interleukin-6 (IL-6), and interleukin-1β (IL-1β) across extracts from various seaweed types (Apostolova et al., 2020). The suppression of NF-κB signaling pathways, which control the expression of inflammatory genes, has been connected to this anti-inflammatory effects (Tunon et al., 2009). The beneficial angiogenic activity of fucoidan is further shown by endothelial cell experiments, which reveal that it promotes vascular tube formation stimulated by FGF-2 and alters integrin expression patterns to support endothelial movement and organization (Senni et al., 2011). Signaling pathways including PI3K/AKT, which serve as essential for endothelial proliferation and neovascularization, have been linked to these pro-angiogenic effects (Abdollah et al., 2023). Composite frameworks made of sodium alginate and gelatin enhanced with fucoidan exhibit excellent biocompatibility and notable reductions in oxidative stress indicators. Mice microglial cells cultured with SaG-Fu structures maintained high survival rates with minimal cytotoxicity after LPS stimulation, showing decreased production of reactive oxygen species (ROS), prostaglandin E (PGE), and nitric oxide (Nguyen et al., 2016). The decrease in oxidative stress indicators emphasizes fucoidan’s antioxidant potential, which helps shield cells from inflammation. Fucoidan’s integrative role in regulating oxidative stress, inflammation, and cellular signaling pathways results in increased biological activity when compared to pure alginate systems (Apostolova et al., 2020). Overall, these results suggest that alginate–fucoidan combinations create a favorable environment for cellular growth and protection against oxidative and inflammatory stress.

## 14 *In vivo* preclinical examination

Alginate–fucoidan combinations have been shown to have therapeutic applications in complex wound environments through extensive preliminary research in animal models. Through the activation of important regenerative signaling pathways, such as VEGF-mediated angiogenesis and TGF-β1-driven collagen synthesis, which are crucial for tissue remodeling, these processes improve wound healing, according to recent *in vivo* research (Lin et al., 2025). Topical application of these mixtures in rats significantly sped up wound healing, stimulated collagen production, and promoted new blood vessel formation by activating VEGF and TGF-β1 signaling pathways (Wang et al., 2025). Designs incorporating fucoidan and silver nanoparticles have demonstrated strong antibacterial and antioxidant properties in diabetic wound models, along with improved tissue regeneration and faster wound closure. These synthetic patches enhanced wound contraction in early tests by reducing bacterial invasion, decreasing oxidative stress markers, and supporting tissue granulation and angiogenesis (Saravanakumar et al., 2024; Hao et al., 2025). Fucoidan-based systems have also been demonstrated to control macrophage differentiation toward the M2 phenotype in diabetic circumstances, which promotes repair of tissues and anti-inflammatory actions and enhances the healing of chronic wounds (Wang et al., 2026).

Burn and bleeding injury types provide additional support for those results. In relation to untreated controls, adding fucoidan to chitosan hydrogels substantially improved wound closure, dermal microvascular repair, and whole skin structure in rabbit burn injuries (Sezer et al., 2008). The AKT/Nrf2/HIF-1α pathways were activated in thickened resection wounds treated with fucoidan, which significantly accelerated tissue repair by enhancing vascular development, collagen deposition, and epithelial regeneration (Wen et al., 2023). These processes are essential for minimizing oxidative stress and encouraging angiogenesis, which improves tissue regeneration in inflammatory circumstances. These outcomes highlight the cooperative role of fucoidan in inflammation resolution, extracellular matrix remodeling, and vascular development during skin reconstruction.

### 14.1 Early-stage clinical evaluations

Although medical research is still in its early stages, early results are promising. The majority of the clinical evidence available now comes from tests and early-phase trials that concentrate on safety, tolerability and initial efficacy in the treatment of chronic wounds (Chilwant et al., 2025). An initial study examined alginate-fucoidan bandages for ulcers caused by diabetes in patients. The treatment sped up cell growth, shortened healing times, and caused no discomfort or allergic reactions (Karma et al., 2024). A key step toward clinical use is also being made with a Phase I/II study currently assessing the safety and effectiveness of fucoidan-based patches for treating chronic vein ulcers (Alfinaikh et al., 2025). Regulatory challenges that include batch-to-batch variability, the absence of standardized extraction methodologies, and the requirement for consistent product characterization to comply with regulatory approval standards continue to limit wider clinical translation despite these encouraging results (Younas et al., 2023; Chadwick et al., 2025).

## 15 Cartilage regeneration

### 15.1 Adipose stem cells embedded in alginate–gelatin microspheres for cartilage restoration

**Figure 6:**
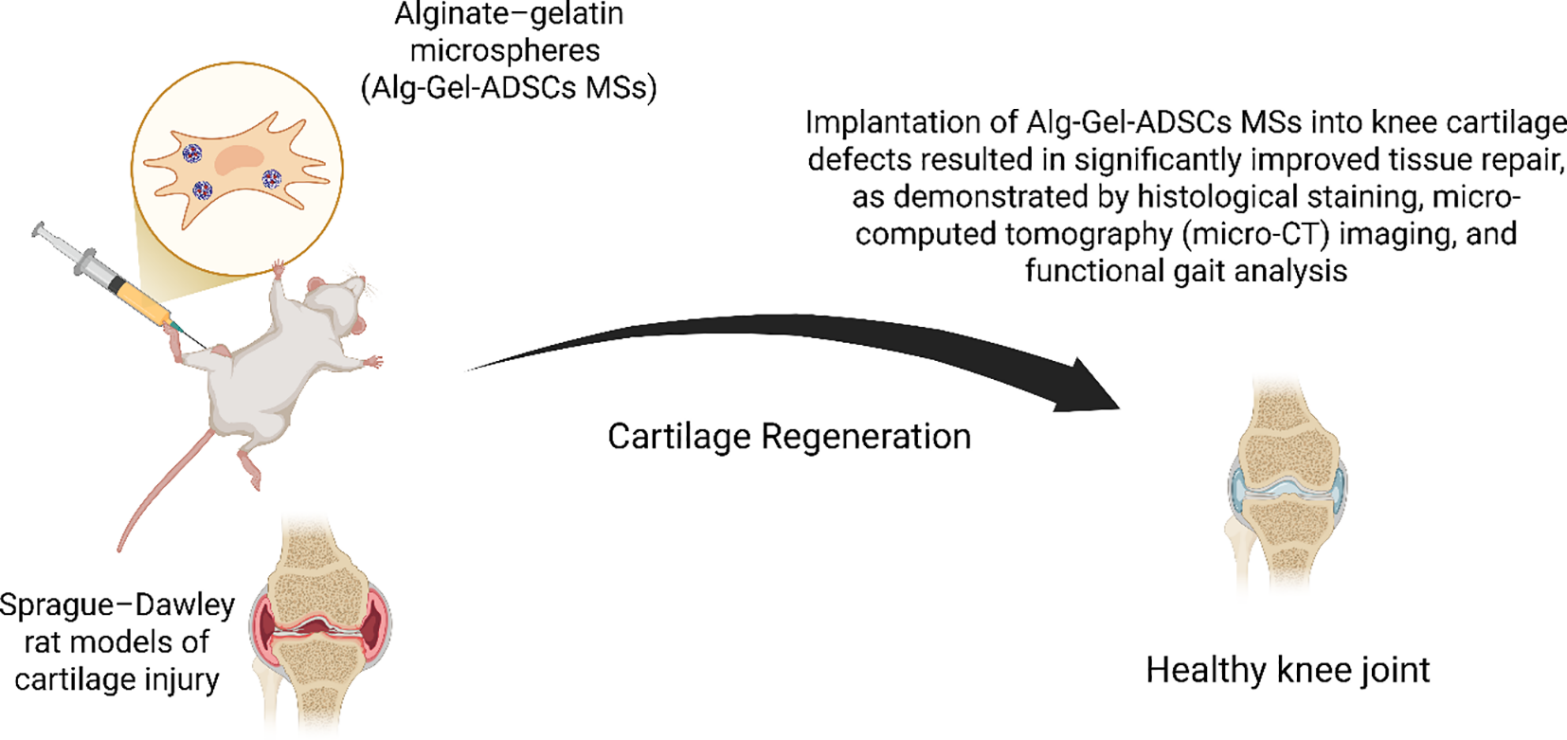
Alginate–gelatin microsphere-delivered ADSCs enhance cartilage repair in rat knee injury models.

To improve cartilage repair, recent research has explored using alginate and gelatin nanoparticles as delivery carriers for adipose-derived stem cells (ADSCs), as shown in **Figure 6**. Liao et al. (2022), employed electrospray technology to create implanted ADSCs with alginate and gelatin nanoparticles (Alg-Gel-ADSCs MSs) and assessed their effectiveness as a treatment in Sprague–Dawley rat models of cartilage damage. The nanoparticles maintained good cell survival, promoted ADSC growth and cartilage formation, and exhibited a consistent, nearly spherical shape. *In vitro*, these combined materials helped enhance extracellular matrix (ECM) deposition and glycosaminoglycan (GAG) synthesis compared to alginate alone. *In vivo*, implantation of Alg-Gel-ADSCs MSs into knee cartilage defects led to significantly improved tissue repair, as shown by histological staining, micro-computed tomography (micro-CT) imaging, and gait analysis. These findings demonstrate that alginate and gelatin nanoparticles create a beneficial three-dimensional environment that boosts stem cell growth potential, offering a promising approach for cartilage regeneration. Additionally, collagen derived from marine sources, chitosan, fucoidan, and chondroitin sulfate have been developed as freezing biomaterials loaded with human primary cells to stimulate new cartilage growth in joints, according to Carvalho et al. (2023),. The inclusion of fucoidan improved these frozen scaffolds in terms of elasticity, injectability, and strength. Furthermore, *in vitro* studies showed that fucoidan-loaded frozen gels support the viability and growth of human adipose-derived stem cells (hADSCs) by providing a suitable habitat. Based on this research, marine biomaterials enhanced with fucoidan show promise as biologically active substrates for cartilage repair and healing.

## 16 Tissue engineering

Because fucoidan and gelatin nanoparticles have multifunctional features such as biodegradability and biocompatibility, they have demonstrated significant potential in clinical use. Different drug delivery platforms, such as polymeric hydrogels, liposomal systems, and synthetic polymer nanoparticles, have been studied in applications in tissue engineering. Even so, these mechanisms frequently have drawbacks including poor biocompatibility, burst release, or lack of biofunctionality (Naderi et al., 2011). Doxorubicin (DOX) was used as an anticancer drug to assess the viability of these nanoparticles as drug delivery vehicles. They show high DOX-loading efficiency and a pH-adaptive release profile, with noticeably higher releases in acidic conditions that resemble the tumor microenvironment, which yields the best results and strongly supports the use of fucoidan with gelatin as nanoparticles for targeted anticancer drug delivery in the study by Ho et al. (2023),. Fucoidan-gelatin systems have extra biological benefits over traditional carriers like PEG-based nanoparticles and synthetic polymer scaffolds, such as increased interaction with growth factors involved in tissue regeneration, greater cell affinity, and intrinsic bioactivity (Ho et al., 2023). The small particles demonstrated regulated biological degradation behavior in addition to their ability as drug delivery, representing that they could be used in temporary immersion chemotherapy for brief periods of time. The effectiveness of these particles as building blocks in tissue engineering is further shown by the essential binding of fucoidan which has ability for binding to heparin proteins and the good properties of gelatin such as elasticity and mechanical support provides these roles. Although uses for tissue engineering were not fully examined in this study, its biological and physicochemical characteristics were reported by Ho et al. (2023),.

## 17 The regeneration of bone tissue (fucoidan-based scaffolds)

Recently, composites including fucoidan have shown promise as biological materials for bone tissue repair. Fucoidan-based biomaterials are interesting applicants for bone tissue engineering applications because, according to several recent research, they greatly improve bone regeneration by encouraging osteogenic differentiation, mineral deposition and extracellular matrix production (Prova et al., 2025). A scaffold made of chitosan, alginate, and fucoidan was created as a potential alternative for reconstructing bone from alveolar cavity restoration. The structure, produced via a freeze-drying process, had a sponge-like appearance with an extremely porous structure essential for cell penetration, circulation, and nutrient absorption (Hamrun et al., 2025). Fucoidan-incorporated hydrogel frameworks and composite structures have shown interest in encouraging osteoblast development and proliferation by enhancing osteogenic factors such osteocalcin, RUNX2, and alkaline phosphatase (ALP). Studies conducted both *in vitro* and *in vivo* have demonstrated that the presence of fucoidan inside the scaffold structure accelerates the production of bone matrix and enhances calcium deposition (Devi GV et al., 2022). Among the tested formulations, which consisted of chitosan, alginate, and fucoidan in a ratio of 1:3:0.1 respectively, exhibited the most desirable structural parameters, with the largest pore size and an absorbency of nearly 86.8%. Additionally, this composition showed a significant reduction in coagulation, demonstrating excellent biological stability and hemocompatibility. For instance, adding fucoidan enhanced the scaffold’s physiological functions by promoting osteoblast development and increasing key proteins linked to bone, such as osteocalcin, collagen type I, and alkaline phosphatase.

## 18 Immunotherapy

**Figure 7:**
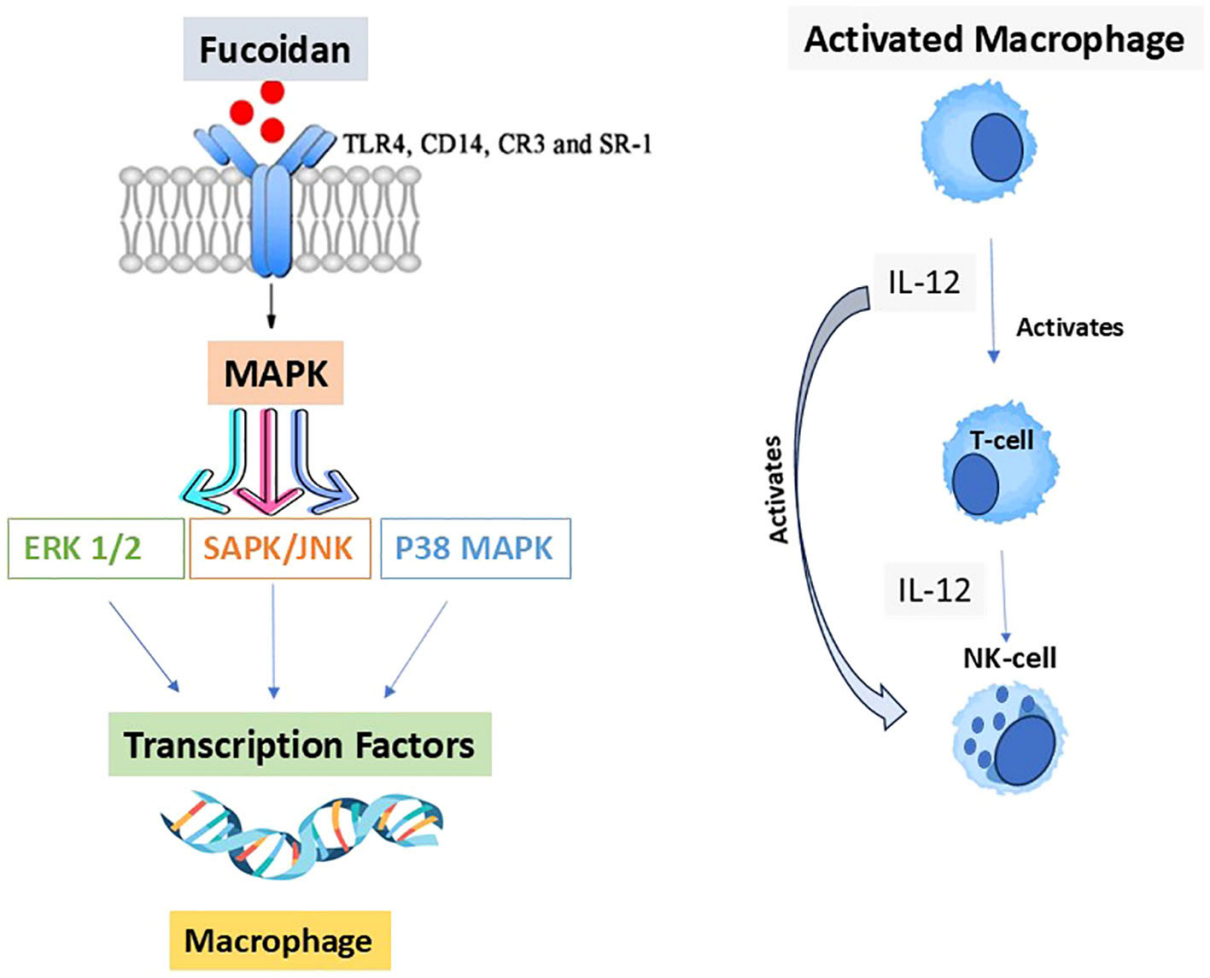
Proposed mechanism of fucoidan (FCSPs) immunomodulatory activity.

The potential of synthetic fucoidan biomaterials to modify immune responses is gaining significant attention lately, especially concerning the immunotherapy of cancer. Fucoidan has natural immune-modulating properties that can alter the tumor environment and enhance immune responses, in addition to serving as structural elements in delivery methods. To act as an *in situ* tumor vaccine, Wang et al. (2024), introduced a novel modular hydrogel composed of fucoidan, chlorin e6, and chloroquine. After photodynamic activation, the hydrogel caused extensive immunogenic cell death, leading to the release of tumor antigens. Fucoidan was crucial in shifting macrophages associated with tumors to the M1 pro-inflammatory phenotype, which boosted innate immune activation. Additionally, chloroquine reduced apoptosis in tumor cells, facilitating better antigen processing and distribution. These combined approaches improved dendritic cell maturation, triggered strong T-cell activation, and effectively prevented tumor growth and metastasis. Further research by (Liu L. et al., 2022) confirmed fucoidan’s immunostimulatory properties. Their study showed that low-molecular-weight fucoidan (LMWF) from *Undaria pinnatifida* enhanced the proliferation of CD4 and CD8 T cells while also promoting dendritic cell maturation and migration to lymphoid tissues. The TLR4–MAPK–NF-κB pathway was the main driver of these immune responses, demonstrating fucoidan’s ability to enhance both innate and adaptive immunity (Liu L. et al., 2022). Fucoidan interacts with macrophage surface receptors, including TLR-4, CD14, CR-3, and SR-1, leading to activation of intracellular MAPK signaling pathways such as ERK1/2, SAPK/JNK, and p38 MAPK. These pathways regulate transcription factors that drive macrophage activation. Activated macrophages then produce cytokines, particularly IL-12, which plays a central role in immune modulation by activating natural killer (NK) cells and T cells. This cascade enhances the overall immune response, with IFN-γ further contributing to the activation and communication between these immune cells as shown in **Figure 7** (Yin et al., 2019). Overall, fucoidan is a versatile biological material that combines carrier functions with natural immune-activating capabilities, as this research clearly shows.

## 19 Renewing medicine

Delivery techniques based on fucoidan have also demonstrated a great deal about the possibilities in the discipline of medical regeneration. The development of injectable GelMA–fucoidan hydrogel microspheres containing Brachyury (Bry) mRNA for the treatment of intervertebral disc degeneration (IVDD) by Díaz-Barrera et al (Gong et al., 2025),. Fucoidan provided strong anti-inflammatory properties to this structure, thereby reducing the inflammatory condition that promotes tissue destruction. Meanwhile, the Bry mRNA supplied improved the functional adaptability of nucleus pulposus cells and promoted the restoration of extracellular matrix (ECM) equilibrium (Gong et al., 2025). These particles successfully promoted ECM remodeling, reduced inflammatory destruction, and slowed the progression of disc degeneration, according to *in vivo* studies in a rat IVDD model. The dual-function feature of fucoidan and hydrogel compounds is demonstrated by the combination anti-inflammatory and regenerating effects.

## 20 Conclusions

Brown seaweeds contain two of the most essential polysaccharides, fucoidan and alginate, which continue to show great potential as environmentally friendly, biodegradable, and ecologically diverse composites. Their physical, chemical, and therapeutic properties are directly influenced by the complexity of their structure, including factors such as the flexible M/G ratio of alginates, molecular weight, and the varied sulfate structures and branches of fucoidan. Advances in extraction techniques, such as enzyme-assisted, microwave-assisted, and ultrasound-assisted methods, have reduced processing time and environmental impact while enhancing productivity and structural stability. However, challenges remain regarding validity, standardized procedures, and batch variability across species and extraction methods. Highly pure fucoidan and alginate can be safely used in various biological systems, as confirmed by biological compatibility studies, and their degradability can be precisely controlled through oxidation, crosslinking, enzymatic processing, and environmental adjustments. These features make them suitable for incorporation into advanced drug delivery systems like hydrogels, nanoparticles, nanofibers, and microsphere-based multidimensional composite structures. Beyond passive carriers, their natural biological properties, including anticoagulant, antioxidant, immunomodulatory, and other beneficial effects, further enhance treatment outcomes. Overall, alginate and fucoidan offer a solid foundation for developing advanced biomedical technologies. To translate experimental findings into therapeutically viable solutions, further efforts are needed in structural characterization, consistency of extraction methods, and optimized material design. Continued advancements in nanotechnology, regenerative medicine, and marine biological processing will cement these polysaccharides as essential materials for producing safe, high-performance, and sustainable biomaterials in the future.
